# Carbazole-Based Colorimetric Anion Sensors †

**DOI:** 10.3390/molecules26113205

**Published:** 2021-05-27

**Authors:** Krystyna Maslowska-Jarzyna, Maria L. Korczak, Jakub A. Wagner, Michał J. Chmielewski

**Affiliations:** Biological and Chemical Research Centre, Faculty of Chemistry, University of Warsaw, Żwirki i Wigury 101, 02-089 Warsaw, Poland; kmaslowska@chem.uw.edu.pl (K.M.-J.); ml.korczak@student.uw.edu.pl (M.L.K.); ja.wagner@student.uw.edu.pl (J.A.W.)

**Keywords:** supramolecular chemistry, anion recognition, anion receptors, anion sensors, colorimetric sensors

## Abstract

Owing to their strong carbazole chromophore and fluorophore, as well as to their powerful and convergent hydrogen bond donors, 1,8-diaminocarbazoles are amongst the most attractive and synthetically versatile building blocks for the construction of anion receptors, sensors, and transporters. Aiming to develop carbazole-based colorimetric anion sensors, herein we describe the synthesis of 1,8-diaminocarbazoles substituted with strongly electron-withdrawing substituents, i.e., 3,6-dicyano and 3,6-dinitro. Both of these precursors were subsequently converted into model diamide receptors. Anion binding studies revealed that the new receptors exhibited significantly enhanced anion affinities, but also significantly increased acidities. We also found that rear substitution of 1,8-diamidocarbazole with two nitro groups shifted its absorption spectrum into the visible region and converted the receptor into a colorimetric anion sensor. The new sensor displayed vivid color and fluorescence changes upon addition of basic anions in wet dimethyl sulfoxide, but it was poorly selective; because of its enhanced acidity, the dominant receptor-anion interaction for most anions was proton transfer and, accordingly, similar changes in color were observed for all basic anions. The highly acidic and strongly binding receptors developed in this study may be applicable in organocatalysis or in pH-switchable anion transport through lipophilic membranes.

## 1. Introduction

Molecules that selectively change their color or fluorescence in the presence of anionic species are highly appealing for applications in various branches of science, industry and medicine [1]. Such changes may be induced by hydrogen bonding interactions with anions, especially in cases where hydrogen bond donors are directly coupled with the receptor’s chromophores or fluorophores [2,3,4,5,6,7]. If these changes are sufficiently strong and affect the visible region of the spectra, they can even be detected by naked eye [2]. Significant research efforts have therefore been directed towards the development of colorimetric and fluorescent anion sensors based on hydrogen bond donating receptors [8,9].

One particularly appealing building block for the development of colorimetric and fluorescent anion sensors is 1,8-diaminocarbazole [10]. It combines several attractive features, such as the presence of a strong carbazole chromophore and fluorophore directly coupled with anion binding sites, a strong hydrogen bond donor (i.e., carbazole NH), a rigid skeleton that facilitates the preorganization of auxiliary hydrogen bond donors and the ease of synthesis and derivatization. Diaminocarbazole-based receptors show particularly high affinity towards oxyanions (carboxylates, phosphates, and sulfates) [3,11,12,13,14,15,16,17,18,19,20,21,22,23,24] and some of them are also very active anion transporters [22,25,26].

**Figure 1:**
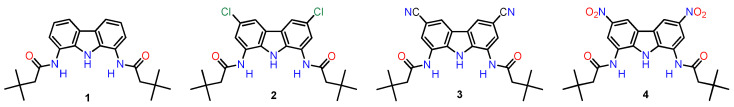
Model receptors investigated in this study.

However, carbazole has negligible absorption in the visible region of the spectrum, and accordingly, in the absence of other chromophores, diaminocarbazole-based receptors are colorless. It has been shown that introducing mildly electron-withdrawing chlorine substituents at positions 3 and 6 shifts the UV-vis spectra of diamidocarbazoles almost to the edge of the visible region, while simultaneously increasing their anion binding constants by up to an order of magnitude [22]. Therefore, we hypothesized that stronger electron-withdrawing substituents could transform such receptors into colorimetric anion sensors and enhance their binding constants even more significantly. In this report we describe the synthesis and anion sensing properties of 3,6-dicyano- and 3,6-dinitro-substituted carbazole receptors (Figure 1; compounds **3** and **4**) and compare them with the previously described [19,22] 3,6-unsubstituted and 3,6-dichlorosubstituted receptors (Figure 1; compounds **1** and **2**).

## 2. Results and Discussion

### 2.1. Synthesis

**Scheme 1:**
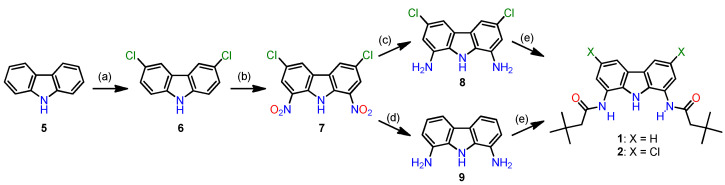
Synthesis of receptors **1** and **2** [19,22]. *Reagents and conditions*: (**a**) SO_2_Cl_2_, CH_2_Cl_2_, RT, 60%. (**b**) HNO_3_ (100%), Ac_2_O/AcOH, 1–110 °C, 73%. (**c**) H_2_ (balloon), 5%Pt(S)/C (cat.), CH_3_CN, RT, 90%. (**d**) NH_2_NH_2_, 10%Pd/C (cat.), EtOH, reflux, 6 h, 75%. (**e**) (CH_3_)_3_CH_2_COCl, Et_3_N, CH_3_CN, RT, 40–88%.

Carbazole is electron rich and readily undergoes electrophilic aromatic substitution at positions 3 and 6. Once these positions are blocked (by chlorine atoms, for example), nitration can occur smoothly at positions 1 and 8 to afford the desired dinitro derivative (i.e., 3,6-dichloro-1,8-dinitrocarbazole (**7**)) [27]. Subsequent reduction yields either 1,8-diamino-3,6-dichlorocarbazole (**8**) [22] or 1,8-diaminocarbazole (**9**) [28], depending upon the reagents and conditions. These two diamines have already been used for the synthesis of various anion receptors, including amides, thioamides, sulfonamides, and ureas [17,18,20,26]. Additionally, our group has used them also for the synthesis of model receptors **1** and **2** (Scheme 1) [19,22].

**Scheme 2:**
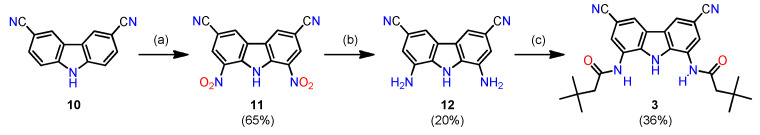
The synthesis of receptor **3**. *Reagents and conditions*: (a) HNO_3_ (100%), Ac_2_O/AcOH, 1 °C to 110 °C, 12 h, 66%; (b) NH_2_NH_2_·H_2_O, FeSO_4_·7H_2_O, EtOH, reflux, 48 h, 20%; (c) (CH_3_)_3_CH_2_COCl, DMA, RT, 24 h, 36%.

A similar strategy has been adopted in the present work for the synthesis of the 3,6-dicyano substituted receptor **3** (Scheme 2).

**Scheme 3:**
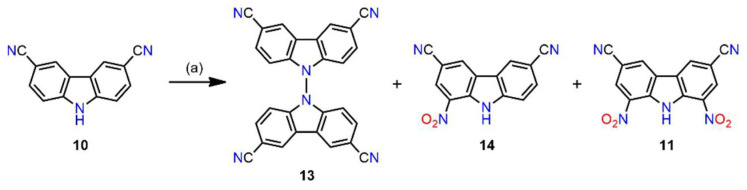
Nitration of 3,6-dicyanocarbazole **10**. *Reagents and conditions*: (a) HNO_3_ (100%)/Ac_2_O/ AcOH, various temperatures.

The synthesis begins with 3,6-dicyanocarbazole (**10**), which is easily obtained in two steps starting from carbazole [29]. The nitration of **10** with acetyl nitrate (formed in situ from 100% nitric acid and acetic anhydride), under conditions optimized for 3,6-dichlorocarbazole **6** [27], led to a 2:1 mixture of the mononitro- and dinitro- derivatives (Scheme 3, compounds **14** and **11**). This is apparently due to the stronger deactivation of the carbazole core by the -CN groups. On the other hand, much harsher conditions, such as boiling in the HNO_3_/Ac_2_O/AcOH mixture or using a mixture of concentrated sulfuric and nitric acids, primarily lead to the formation of degradation products or 1,3,6,8-tetranitrocarbazole **15**, respectively. Therefore, the reaction conditions were carefully optimized, particularly in terms of the temperature and the substrate/HNO_3_ and HNO_3_/Ac_2_O ratios.

Surprisingly, at 50 °C the main product was *N*,*N’*-bis(3,6-dicyanocarbazole) (**13**), and only traces of nitrated products were detected in the reaction mixture after 40 h (Scheme 3). Heating at 70 °C for 40 h led to a mixture of biscarbazole **13**, 3,6-dicyano-1-nitrocarbazole (**14**) and a small amount of the desired 3,6-dicyano-1,8-dinitrocarbazole (**11**). Further increasing the reaction temperature to 90 °C resulted in the complete disappearance of **13** and preferential formation of the desired dinitrocarbazole **11**; however, a significant amount of the mononitro derivative **14** was still present in the reaction mixture under these conditions. The two nitro compounds were very difficult to separate, and therefore, it was necessary to further optimize the reaction conditions to avoid the formation of this by-product. Ultimately, at 110 °C, very clean formation of the dinitro product was observed. This was somewhat surprising, given that at 118 °C (the boiling point of the mixture) extensive decomposition occurred. Additionally, the nitrating reagent (i.e., acetyl nitrate) decomposes more and more rapidly at temperatures above 60 °C [30]. Consequently, nitration at 110 °C requires a large excess of 100% HNO_3_ and is quite capricious.

To circumvent the extensive decomposition of acetyl nitrate, the nitric acid was added in small portions, and the mixture was cooled down prior to each addition. The reaction was monitored by ^1^H NMR and allowed to continue until all the signals corresponding to **14** disappeared. Using these precautions, clean formation of **11** was achieved. The product precipitated from the reaction mixture in an almost pure form and could be obtained in 66% yield after simple filtration and washing with diethyl ether. It is worth noting that the 3,6-dicyano-1,8-dinitrocarbazole (**11**) may be a very useful synthon because it bears up to five amino group equivalents on a small, rigid core and could be produced in just three steps from the very cheap starting material, carbazole.

Selective reduction of the two nitro groups in **11** proved difficult, and this reaction gave the desired 1,8-diamino-3,6-dicyanocarbazole (**12**) in only 20% yield (not optimized). Additionally, the final acylation of **12** with *t*-butylacetyl chloride was more problematic than in the case of receptors **1** and **2**, because the strongly electron withdrawing -CN groups rendered **3** prone to over-acylation, which generated an imide impurity that was very difficult to separate. Therefore, it was necessary to develop a modified protocol for this reaction. It was determined that in the absence of any base and in dimethylacetamide (DMA) as a solvent, the imide formation was suppressed, and the desired receptor **3** could be obtained in its pure form, although in only 36% yield (Scheme 2).

**Scheme 4:**
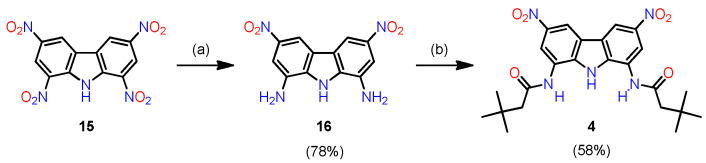
Synthetic pathway leading to the receptor **4**. Isolated yields are given in parentheses. *Reagents and conditions*: (**a**) NH_2_NH_2_·H_2_O, FeSO_4_·7H_2_O, EtOH, reflux, 20h; (**b**) 3,3-dimethylbutyryl chloride, DMA, RT, 20h.

Extending the same synthetic strategy to the synthesis of receptor **4** was problematic because it required the selective reduction of only two of the four nitro groups in 1,3,6,8-tetranitrocarbazole **15** (Scheme 4). However, since the tetranitrocarbazole **15** was commercially available, we took up the challenge and, after some experimentation, found the appropriate reagents and conditions for this transformation. Specifically, 1,8-diamino-3,6-dinitrocarbazole (**16**) could be obtained selectively in 78% yield using hydrazine hydrate as the reductant and iron(II) sulfate as the catalyst [31]. This finding enabled convenient access to another very attractive synthon, which also bears five amino group equivalents on a single carbazole core.

The acylation of **16** with *t*-butylacetyl chloride under standard conditions (acetonitrile, Et_3_N) also produced a difficult to separate imide impurity. As in the case of **3** described above, elimination of any base and use of DMA as the solvent suppressed the over-acylation and yielded pure receptor **4** in 58% yield.

### 2.2. Anion Recognition Studies

Anion binding studies with receptors **1** and **2** have been described previously [19,22]. To allow for comparisons, the interactions of anions with receptors **3** and **4** were studied using identical methods and conditions.

**Figure 2:**
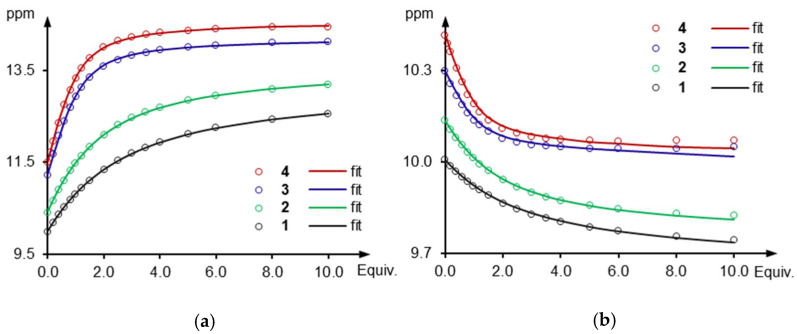
^1^H NMR titration curves of the (**a**) NH_carb._ and (**b**) NH_amide_ signals of receptors **1**–**4** (10 mM) for TBACl in DMSO-d_6_ + 0.5% H_2_O at 298 K. The 1:1 modeled fits are shown as solid lines.

The results of ^1^H NMR titrations of receptors **3** and **4** with tetrabutylammonium (TBA) chloride in deuterated dimethyl sulfoxide (DMSO-d_6_) + 0.5% H_2_O are presented in Figure 2, together with the results for receptors **1** and **2**. Comparing the chemical shifts of free receptors **1**-**4** revealed the substantial influence of the rear substituents, X, on the position of the NH protons. The carbazole NH signal shifted from 9.99 ppm for X=H, to 10.41 ppm for X=Cl, 11.22 ppm for X=CN, and 11.49 ppm for X=NO_2_. Similar but smaller downfield shifts (from 9.97 for **1** to 10.39 for **4**) were observed for the amide NHs. Therefore, as the electron-withdrawing character of the 3,6-substituents increased, the two NH protons became more and more strongly engaged in hydrogen bonding interactions with solvent molecules.

The addition of tetrabutylammonium chloride (TBACl) to solutions of the four receptors resulted in large downfield shifts of the carbazole NH protons (Δδ_max_ = 2.56, 2.79, 2.89 and 2.98 ppm, for **1**–**4**, respectively) and slight upfield shifts of the amide NH protons (Δδ_max_ = −0.27, −0.32, −0.28, and −0.37 for **1**–**4**, respectively). These results indicated that the hydrogen bonds formed between the anion and the central carbazole NH groups were much stronger than those formed with the two amide side arms. The rather uncommon upfield shifts exhibited by the amide protons in this family of receptors upon chloride binding suggested that strong hydrogen bonding interactions with solvent molecules deshield these protons more than the hydrogen bonding with chloride. Interestingly, a significant (ca. 0.5 ppm) downfield shift was observed for protons C2-H and C7-H in all four chloride complexes. This is most likely due to locking of the two amide arms in the *syn*-*syn* conformation upon anion binding. This anion-induced conformational change places the amide carbonyl groups in close proximity to the protons C2-H and C7-H, which allows for H-bond type of interaction between the carbonyl oxygens and these protons leading to their downfield shift.

**Figure 3:**
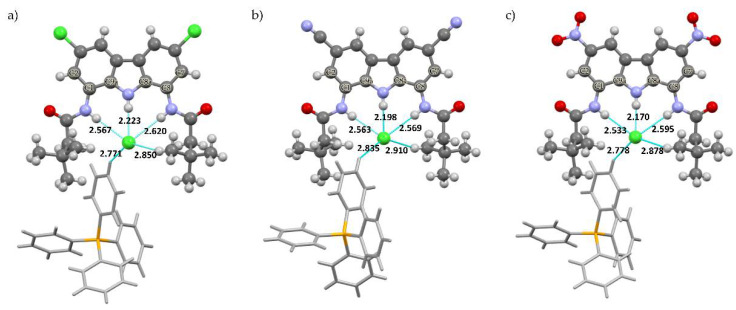
Crystals structures of (**a**) **2** × Cl^−^, (**b**) **3** × Cl^−^ and (**c**) **4** × Cl^−^.

These conclusions were supported by X-ray crystal structure analysis of the chloride complexes of diamides **2**, **3** and **4**, in which the carbazole NH–Cl distances were much shorter (2.223, 2.198 and 2.170 Å, respectively) than the amide NH–Cl distances (2.533–2.620 Å), as presented in Figure 3 and Table 1. The asymmetric position of chloride in the cavity of **4** also suggested that while the anion was able to form shorter bonds with the amide group (2.533 Å), the cavity of the receptor was too wide to allow for the simultaneous formation of two short bonds with this relatively small guest. Therefore, it is very likely that in solution, the anion rapidly jumps between two equivalent positions, closer to one or the other amide arm. Since the observed amide proton chemical shifts were averaged over all conformations (fast exchange regime), this hypothesis explains the rather weak effect of chloride binding on the chemical shift of amide protons.

Quantitative analysis of the titration curves was performed using the web applet, BindFit [32]. Excellent fits were obtained using a simple 1:1 binding model, and the association constants thus obtained are presented in Table 2. As expected, introducing strongly electron-withdrawing cyano substituents at positions 3 and 6 enhanced the chloride affinity more than six-fold with respect to the unsubstituted **1** and approx. two-fold with respect to chlorine-substituted **2**. Substitution with even stronger electron-withdrawing nitro groups further increased the Cl^−^ affinity, with the binding constant reaching a value seven times higher than for the unsubstituted receptor **1**.

**Figure 4:**
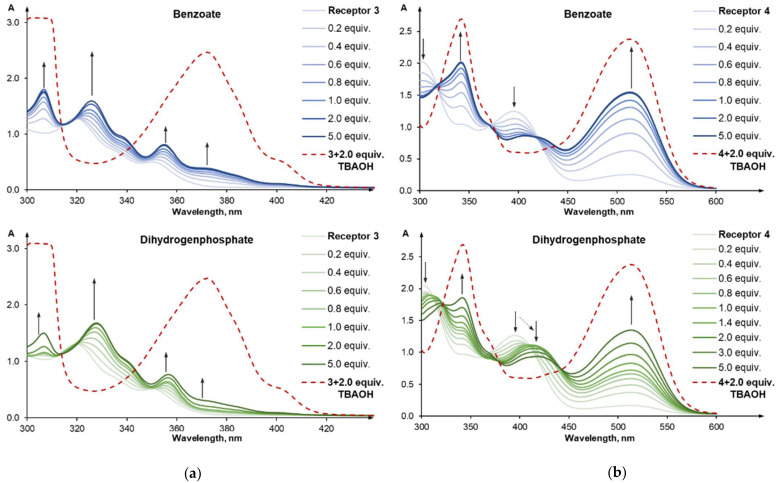
UV-vis titration of: (**a**) **3** (10^−4^ M) with TBAPhCOO (top) and TBAH_2_PO_4_ (bottom); (**b**) **4** (10^−4^ M) with TBAPhCOO (top) and TBAH_2_PO_4_ (bottom) in DMSO + 0.5% H_2_O at 298 K.

The binding constants of 1,8-diamidocarbazoles with benzoate and dihydrogen phosphate typically exceed the range that can be reliably determined by NMR titrations. Fortunately, owing to the carbazole chromophore, this class of receptors is perfectly suited for studying anion binding processes using UV-vis spectroscopy. The addition of oxyanions (as TBA salts) to the solutions containing receptors **3** and **4** in DMSO + 0.5% H_2_O led to clear changes in absorbance between 300 and 600 nm (Figure 4).

However, the absence of well-defined isosbestic points and the formation of characteristic peaks at 372 nm (for **3**) and at 515 nm (for **4**), suggested that the observed changes were not only due to anion binding, but also influenced by proton transfer from the receptors to the basic anions. In the case of **3** and H_2_PO_4_^−^, the proton transfer became significant only after most of the receptor molecules had bound with anions; this enabled an estimation of the anion binding constant from the truncated titration curve. The obtained value, logK = ~5.4, was consistent with those obtained earlier for **1** and **2** (Table 2) and with the expected increase in anion affinity with increasing electron-withdrawing character of the **3**, **6** substituents. Furthermore, it was supported by the observed rapid saturation of the complexation-induced changes in the UV-vis spectra, which occurred following the addition of only 1.6 equivalents of H_2_PO_4_^−^ (in the presence of 10^−4^ M **3**). In the case of **4**, however, the proton transfer dominated the anion binding, and all attempts to separate the two processes were unsuccessful.

### 2.3. Self-Dissociation Studies

**Scheme 5:**
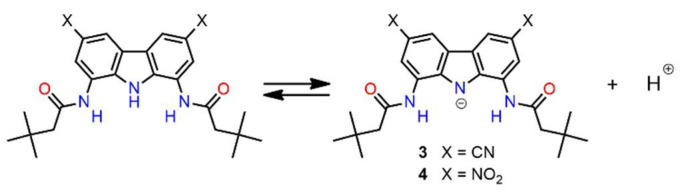
Self-ionization of receptors **3** and **4**.

**Figure 5:**
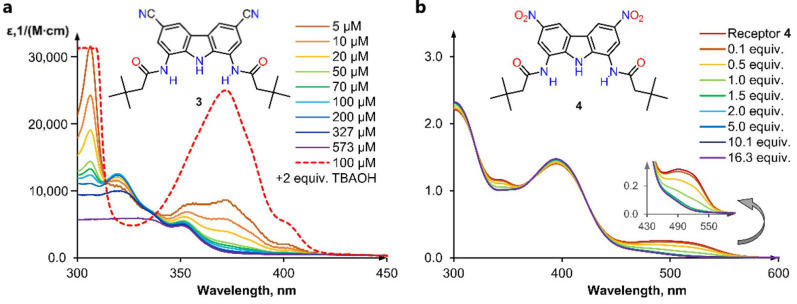
Self-ionization studies. (**a**) Self-ionization of diluted solutions of **3** in DMSO + 0.5% H_2_O at 298 K; (**b**) reversal of self-ionization of **4** (10^−4^ M) upon titration with TfOH in DMSO + 0.5% H_2_O at 298 K.

Careful analysis of the UV-vis titration data presented above revealed that receptors **3** and **4** were slightly deprotonated even before the addition of any anion (Figure 5). Indeed, further dilution of the receptors below the 10^−4^ M concentration used in the titration experiments notably increased the extent of self-ionization (Scheme 5), in accordance with the Ostwald’s dilution law.

**Figure 6:**
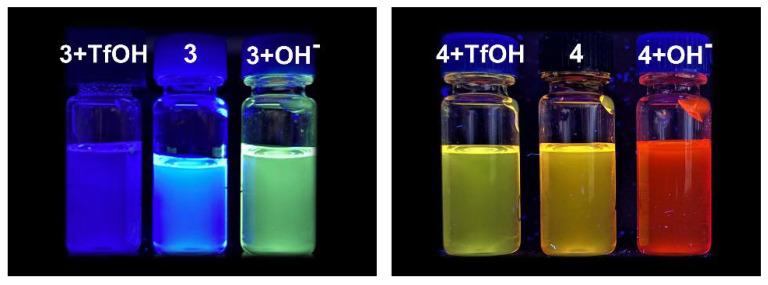
Fluorescence color changes of **3** (left) and **4** (right) at 10^−4^ M in the presence of 2 equiv. of TfOH or 2 equiv. of TBAOH in DMSO + 0.5% H_2_O observed under a UV lamp (365 nm).

**Figure 7:**
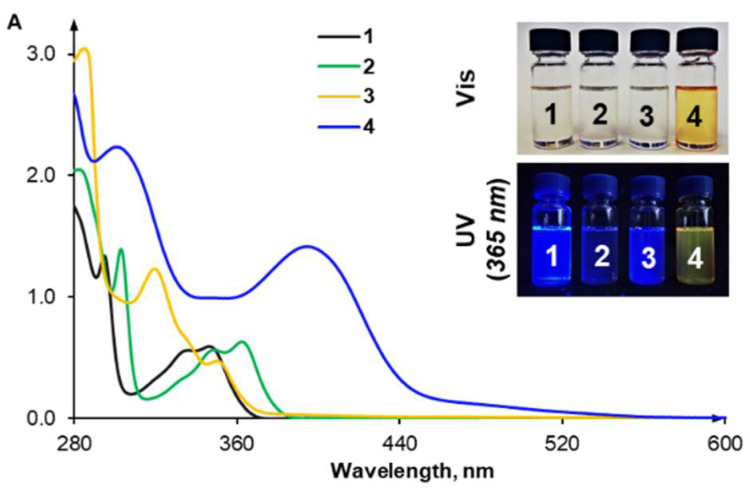
UV-vis spectra of receptors **1**–**4 (**10^−4^ M) in the presence of 2 equiv. of TfOH in DMSO + 0.5% H_2_O at 298 K. Inset: the same solutions under visible light and under UV irradiation (365 nm).

At 5 × 10^−6^ M, even the less acidic **3** was completely ionized (Figure 5a). Titration with trifluoromethanesulfonic acid (TfOH) reversed the self-ionization of both receptors (see ESI 3.1 for **3** and Figure 5b for **4**). It is worth noting that even a very small degree of spontaneous dissociation may lead to erroneous conclusions about the color, fluorescence, and anion sensing properties of receptors (see, for example, Figure 6). Therefore, we present UV-vis spectra and photographs of solutions of **3** and **4** in the presence of two equivalents of TfOH (Figure 7).

### 2.4. Anion Sensing Properties of 3 and 4

As shown in Figure 7, the introduction of nitro groups successfully shifted the absorbance spectrum of **4** into the visible region (>400 nm). Accordingly, both **4** and its solution were intensely yellow. However, a similar effect was not observed when cyano groups were introduced, and the long wavelength region of the spectrum vanished even faster for **3** than for **2**.

**Figure 8:**
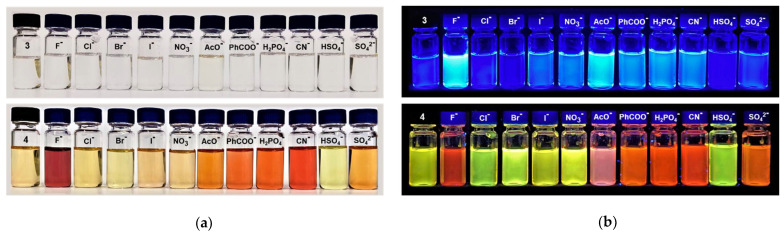
Color changes of 10^−4^ M solutions of **3** and **4** in the presence of 10 equiv. of various anions (added as TBA salts) in DMSO + 0.5% H_2_O: (**a**) under ambient light and (**b**) under a UV lamp (365 nm).

To investigate the sensing capabilities of **3** and **4**, we added various anions were added (as TBA salts) to solutions of each receptor in DMSO + 0.5% H_2_O (Figure 8). Anions with different shapes, solvation energies, and basicity were tested: F^−^, Cl^−^, Br^−^, I^−^, NO_3_^−^, AcO^−^, PhCOO^−^, H_2_PO_4_^−^, CN^−^, HSO_4_^−^ and SO_4_^2−^.

No color change was observed for **3** because of the very weak absorption of **3** and its complexes in the visible region (Figure 4a). However, much more significant changes were observed under UV irradiation (λ_ex_ = 365 nm). A significant enhancement in fluorescent emission was clearly visible in the samples containing the most basic anions, i.e., F^−^, AcO^−^, PhCOO^−^, H_2_PO_4_^−^, and CN^−^, because the deprotonated form of **3** was strongly emissive in the visible region. Fluorescence quenching was observed in the case of HSO_4_^−^, most likely because this anion acted like a Brønsted acid and reversed the spontaneous dissociation of **3**, thereby diminishing the concentration of the highly-emissive [**3** − H^+^]^−^.

In contrast, receptor **4** displayed a clear colorimetric response in the presence of F^−^, AcO^−^, PhCOO^−^, H_2_PO_4_^−^, CN^−^ and SO_4_^2-^, changing color from yellow to orange or burgundy (Figure 8a). Almost no change was observed upon addition of Cl^−^, Br^−^, I^−^, NO_3_^−^, or HSO_4_^−^. Since the colorimetric response was only observed for basic anions and was similar for all of them, the phenomenon was most likely dominated by the deprotonation of the receptor. As discussed above, both anion binding and receptor deprotonation shifted the absorption spectra of **3** and **4** toward longer wavelengths, and both processes coexisted for PhCOO^−^ and H_2_PO_4_^−^ in wet DMSO. However, in case of the more acidic **4**, proton transfer dominated, and this was further amplified by the fact that the extinction coefficients of [**4** − H^+^]^−^ were much higher than of [**4** × A^−^]^−^. As a result, the colorimetric response of **4** exhibited low specificity, i.e., it was very similar for all basic anions. Analogous behavior has often been observed in hydrogen bonding anion sensors [9].

The above conclusions gained additional support from the anion-induced changes in the fluorescence of **4**. Under UV irradiation (365 nm), dramatic changes in emission from yellow-green to orange-red were observed upon addition of basic anions, i.e., F^−^, AcO^−^, PhCOO^−^, H_2_PO_4_^−^, CN^−^ and SO_4_^2−^, whereas little or no change was induced by Cl^−^, Br^−^, I^−^, NO_3_^−^, or HSO_4_^−^. Again, the weakly-basic and weakly-binding anions in the latter group caused negligible changes, whereas all of the anions in the former group deprotonated **4** to a certain extent (slight or significant), thus switching the greenish-yellow fluorescence of the receptor into the orange-red fluorescence of the deprotonated receptor. Unlike in the case of **3**, the doubly-charged sulfate anion could deprotonate **4**, in accordance with the increased acidity of this receptor.

Unfortunately, more detailed spectrofluorimetric investigations of **3**, **4**, and their interactions with anions were hindered by the spontaneous dissociation of the receptors in extremely diluted solutions, such as those typically used for fluorescence measurements.

## 3. Materials and Methods

### 3.1. General Experimental

#### 3.1.1. Chemicals and Consumables

All solvents and reagents were commercially available and used as received unless otherwise stated. Reagents for this study were purchased from the following vendors:

- Merck-Sigma (Darmstadt, Germany): acetone (puriss. p.a., ≥99.5% (GC), 32201), acetonitrile (ACN; ≥99.9%, 34998), *N*,*N*-dimethylacetamide (DMA; anhydrous, 99.8%, 271012), 3,3-dimethylbutyryl chloride (*tert*-butylacetyl chloride; 99%, B88802), *N*,*N*-dimethylformamide (DMF; puriss. p.a., ACS reagent, reag. Ph. Eur., ≥99.8% (GC), 33120), dimethyl sulfoxide (DMSO; anhydrous, ≥99.9%, 276855), hydrazine monohydrate (N_2_H_4_·H_2_O, reagent grade, 98%, 207942), iron(II) sulfate heptahydrate (FeSO_4_·7H_2_O, ≥99.0%, 215422), methanol (MeOH; HPLC grade, ≥99.9%, 34860), nitric acid (fuming HNO_3_; extra pure, 100%, 1004551000), sodium hydroxide solution (2M NaOH, Titripur, 1091361000), tetrabutylammonium chloride (TBACl; ≥99.0%, 86852), tetrabutylammonium benzoate (TBAPhCOO; ≥99.0%, 86837), tetrabutylammonium phosphate monobasic (TBAH_2_PO4; ≥99.0% (T), 86833).
- POCH S.A. (Gliwice, Poland): acetic acid (AcOH; 99.5-99.9%, pure p.a.-basic, BA8760114), acetic anhydride (Ac_2_O; ACS reagent, pure p.a., 693870115), ethyl alcohol (EtOH; anhydrous, 99.8% pure p.a., 396480111), nitric acid (HNO_3_; 65%, pure p.a., BA9603115), hydrochloric acid (HCl; 35-38%, pure p.a., BA5283115).
- TCI (Zwijndrecht, Belgium): 1,3,6,8-tetranitrocarbazole (wetted with ca. 40% water, >70.0%, T0159).
- Alfa Aesar (Haverhill, MA, United States): trifluoromethanesulfonic acid (TfOH, anhydrous, 98%).
- Euriso-top (Saint-Aubin, France): DMSO-d_6_ + 0.03%TMS *v*/*v* (>99.8% D).
- Linegal Chemicals (Blizne Łaszczyńskiego, Poland): dichloromethane (DCM, pure p.a., 50-8124.4), freshly distilled over CaH_2_.

Water was obtained from a Milli-Q purification system. DMSO/H_2_O mixtures were prepared using Milli-Q H_2_O, and their concentrations are expressed as weight-weight percentages.

#### 3.1.2. Instruments and Methods

Samples were weighed on an Excellence XA105DU analytical balance (Mettler Toledo, Warszawa, Poland). High-resolution electrospray ionization-mass spectrometry (ESI-MS) data were obtained using a MALDI SYNAPT G2-S HDMS mass spectrometer (Waters, Milford, MA, USA) with direct fusion. Elemental analysis was performed using an automatic UNIcube elemental analyzer (Eltra Elemental Analyzers, Warszawa Poland). UV-vis spectra were obtained on an Evolution 300 UV-vis spectrophotometer (Thermo Scientific, Waltham, MA, USA). NMR spectra were recorded using an Agilent NMR (^1^H: 400 MHz; ^13^C: 100 MHz) spectrometer (Agilent Technologies, Santa Clara, CA, USA) at ambient temperature, in DMSO-d_6_. The chemical shifts (δ) are reported in parts per million (ppm), and the coupling constants (*J*) are given in hertz (Hz). The NMR spectra were referenced to the solvent residual signal (^1^H: δ_DMSO_ = 2.50 ppm, ^13^C: δ_DMSO_ = 39.50 ppm). Data are reported as follows: chemical shift, multiplicity (s—singlet, bs—broad singlet, d—doublet, t—triplet, dd—doublet of doublets, dt—doublet of triplets, etc.), coupling constant and integration.

### 3.2. Synthesis

#### 3.2.1. General Methods

Thin layer chromatography (TLC) was carried out on Merck silica gel 60 F_254_ plates. Preparative chromatography was performed using a CombiFlash system (Teledyne Isco, Lincoln, NE, USA) equipped with RediSep Normal-phase Silica Flash Columns. The compositions of eluent mixtures used for chromatographic separations are reported as (*v/v*) ratios.

#### 3.2.2. Synthesis of the previously-reported receptors **1** and **2**

Receptor **1** was obtained in one-step from 1,8-diaminocarbazole [28], as described previously [22]. Receptor **2** was obtained in one-step from 1,8-diamino-3,6-dichlorocarbazole [22], as described previously [19].

#### 3.2.3. Synthesis of Receptor **3**

Receptor **3** was obtained in a three-steps synthesis from 3,6-dicyjanocarbazole **10** [29].

##### 3,6-Dicyano-1,8-dinitrocarbazole (**11**)

To a 50 mL round-bottom two-neck flask equipped with a magnetic stirrer, 3,6-dicyanocarbazole (0.652 g, 3.00 mmol), acetic anhydride (10 mL, 0.11 mol), and acetic acid (13 mL, 0.23 mol) were added. The flask was equipped with a reflux condenser connected to a check-valve bubbler, and 100% nitric acid (0.79 mL, 19 mmol) was added drop-by-drop through the side neck of the flask using a Pasteur pipette. The neck was closed tightly with a glass stopper, and the mixture was heated with stirring to 110 °C (measured in an oil bath). After 50 min (from the beginning of the heating), the reaction mixture was cooled down for 10 min to RT, and another portion of 100% nitric acid (0.79 mL, 19 mmol) was added. The mixture was heated again to 110 °C, and this procedure was repeated four times; 3.16 mL (76 mmol) of nitric acid were added in total. The addition of the fifth portion of 100% nitric acid (0.79 mL, 19 mmol) was preceded by the addition of acetic anhydride (2 mL, 21 mmol). This procedure was repeated five times; 10 mL (105 mmol) of acetic anhydride and 3.95 mL (95 mmol) of nitric acid were added in total. After the eighth addition of HNO_3_/HNO_3_+Ac_2_O, the progress of the reaction was checked by ^1^H NMR, and additional portions of HNO_3_+Ac_2_O were added until the signals corresponding to 3,6-dicyano-1-mononitrocarbazole disappeared. After cooling the reaction mixture to RT, 50 mL of deionized water were added. The precipitate was filtered off and washed with water (2 × 15 mL), MeOH (2 × 5 mL) and diethyl ether (2 × 5 mL). Vacuum drying yielded the pure product as a yellow powder (0.609 g, 66%). ^1^H NMR (400 MHz, DMSO-d_6_) δ_DMSO_: 11.79 (s, 1H, NH_carb._), 9.30 (d, *J* 1.43, 2H, CH4/5 or CH2/7), 8.98 (d, *J* 1.41, 2H, CH4/5 or CH2/7); ^13^C-NMR (101 MHz, DMSO-d_6_) δ_DMSO_: 135.23, 133.35; 133.07, 127.92, 125.26, 117.49, 104.32; HR MS (ESI): *m/z* calcd. for C_14_H_4_N_5_O_4_ [M − H]^−^: 306.0269, found: 306.0263.

##### 1,8-Diamino-3,6-dicyanocarbazole (**12**)

To a 50 mL round-bottom two-neck flask equipped with a magnetic stirrer, 3,6-dicyano-1,8-dinitrocarbazole (0.614 g, 2.00 mmol) and anhydrous EtOH (30 mL) were added. The flask was equipped with a reflux condenser connected to a check-valve bubbler, the side neck was sealed with a rubber septum, and argon was bubbled through the mixture for 10 min with slow stirring. After this time, FeSO_4_·7H_2_O (37 mg, 0.13 mmol) was added, and argon was bubbled through the mixture for an additional 15 min. Then, in the flow of argon, 98% hydrazine monohydrate NH_2_NH_2_·H_2_O (3.90 mL, 80 mmol) was added dropwise. The mixture was heated to reflux and stirred for 48h. After cooling to RT, the precipitate was filtered off and suspended in DMF (ca. 7 mL). The product dissolved in DMF and was separated from insoluble impurities by filtration. Next, methanol (60 mL) was added to the filtrate, and the precipitated impurities were filtered off again using a G4 sintered glass filter. Finally, deionized water (40 mL) containing a few droplets of hydrazine monohydrate (to protect the product from oxidation in air) was added to the filtrate in order to precipitate the desired product. The suspension was boiled for 10 min, cooled to RT, and placed in a refrigerator (at 4 °C) for 24h. The product was filtered off and washed with water (3 × 3 mL). Vacuum drying yielded a product that was sufficiently pure for the subsequent reaction (brown powder, 0.096 g, 20%). ^1^H NMR (400 MHz, DMSO-d_6_) δ_DMSO_: 11.46 (s, 1H, NH_carb_), 7.94 (d, *J* 1.36, 2H, CH4/5 or CH2/7), 6.95 (d, *J* 1.47, 2H, CH4/5 or CH2/7), 5.58 (s, 4H, NH_2_); ^13^C NMR (101 MHz, DMSO-d_6_) δ_DMSO_: 135.23, 133.35, 133.07, 127.92, 125.26, 117.49, 104.32; HR MS (ESI): *m/z* calcd. for C_14_H_8_N_5_ [M − H]^−^: 246.0785, found: 246.0780.

##### Receptor **3**

A 5 mL round-bottom flask was dried with a heat gun (at ca. 500 °C) for 10 min and then cooled to RT in a desiccator. To the dried flask, 1,8-diamino-3,6-dicyanocarbazole (0.050 g, 0.20 mmol) and a magnetic stirrer were added. The neck of the flask was sealed with a rubber septum, and the flask was filled with argon via three pump-thaw cycles. Anhydrous dimethylacetamide (3 mL) was added using a syringe, followed by the addition of 3,3-dimethylbutyryl chloride (0.090 mL, 0.64 mmol). The reaction mixture was stirred at RT for 20h. After this time, the reaction mixture was poured into deionized water (25 mL). The precipitate was filtered off and washed with water (3 × 3 mL), 5% MeOH/DCM (2 × 3 mL) and acetonitrile (ACN; 3 × 3 mL). The crude product was purified by column chromatography on 30 g of silica gel using 3% MeOH/DCM as the eluent. The progress of the chromatographic separation was monitored with TLC using 60% EtOAc/Hexane as an eluent. Fractions containing pure product were combined and the solvent was evaporated to yield 0.032 g (36%) of the desired product as a brown solid. ^1^H NMR (400 MHz, DMSO-d_6_) δ_DMSO_: 11.23 (s, 1H, NH_carb_), 10.26 (s, 2H, NH_amide_), 8.54 (d, *J* 1.3, 2H, CH2/7 or CH4/5), 7.97 (d, *J* 1.12, 2H, CH2/7 or CH4/5), 2.38 (s, 4H, CH_2_) 1.10 (s, 18H, *t*-Bu); ^13^C NMR (101 MHz, DMSO-d_6_) δ_DMSO_: 170.77, 134.56, 124.36, 123.71, 122.45, 122.15, 119.60, 102.26, 49.03, 30.98, 29.64; HR MS (ESI): *m/z* calcd. for C_26_H_29_N_5_O_2_Na [M + Na]^+^: 466.2213, found: 466.2219.

#### 3.2.4. Synthesis of Receptor **4**

Receptor **4** was obtained in two steps from commercially available 1,3,6,8-tetranitrocarbazole.

##### 1,8-Diamino-3,6-dinitrocarbazole (**16**)

Prior to the synthesis, the commercially available 1,3,6,8-tetranitrocarbazole (wetted with ca. 40% water) was dried in a vacuum desiccator over KOH to a constant mass. 1,3,6,8-Tetranitrocarbazole (0.200 g, 0.58 mmol) and anhydrous EtOH (10 mL) were added to a 25 mL round-bottom two-neck flask equipped with a magnetic stirrer. The flask was equipped with a reflux condenser connected to a check-valve bubbler, the side neck was sealed with a rubber septum, and argon was bubbled through the mixture for 10 min with slow stirring. After this time, FeSO_4_·7H_2_O (10 mg, 0.036 mmol) was added, and argon was bubbled through the mixture for additional 15 min. Then, in the flow of argon, 98% NH_2_NH_2_·H_2_O (1.20 mL, 25 mmol) was added dropwise. The mixture was heated to reflux and stirred for 20 h. After cooling to RT, the precipitate was filtered off, sonicated with MeOH for 10 min, and filtered off once again. Vacuum drying yielded the product as a dark brown powder (0.130 g, 78%). The product was used for the subsequent reaction without any additional purification. ^1^H NMR (400 MHz, DMSO-d_6_) δ_DMSO_: 11.71 (s, 1H, NH_carb_), 8.58 (s, 2H, CH4/5 or CH2/7), 7.62 (s, 2H, CH4/5 or CH2/7), 5.76 (s, 4H, NH_2_); ^13^C NMR (101 MHz, DMSO-d_6_) δ_DMSO_: 142.07, 134.70, 133.05, 122.68, 106.74, 103.80; HR MS (ESI): *m/z* calcd. for C_12_H_8_N_5_O_4_ [M − H]^−^: 286.0576, found: 286.0565.

##### Receptor **4**

A 25 mL round-bottom flask was dried with a heat gun (at ca. 500 °C) for 10 min and then cooled down to RT in a desiccator. To the flask, 1,8-diamino-3,6-dinitrocarbazole (0.190 g, 1.00 mmol) was added, followed by a magnetic stirrer. The neck of the flask was sealed with a rubber septum, and the flask was filled with argon via three pump-thaw cycles. Anhydrous dimethylacetamide (10 mL) was added using a syringe, followed by the addition of 3,3-dimethylbutyryl chloride (0.420 mL, 3.00 mmol). The reaction was stirred at RT for 20 h. After this time, the reaction mixture was poured into deionized water (100 mL), cooled down in a refrigerator (at 4 °C) for 20 min, and the precipitate was filtered off and washed with water (3 × 3 mL) and MeOH (2 × 3 mL). The obtained, slightly contaminated product was suspended in acetone (80 mL), sonicated for 10 min, and filtered off. Vacuum drying yielded the pure product as a yellow powder (0.280 g, 58 %). ^1^H NMR (400 MHz, DMSO-d_6_) δ_DMSO_: 11.49 (s, 1H, NH_carb_), 10.38 (s, 2H, NH_amide_), 9.28 (d, *J* 2.2, 2H, CH4/5), 8.55 (d, *J* 2.1, 2H, CH2/7), 2.40 (s, 4H, CH_2_) 1.11 (s, 18H, *t*-Bu); ^13^C NMR (101 MHz, DMSO-d_6_) δ_DMSO_: 170.90, 141.17, 136.07, 123.85, 123.77, 114.54, 114.53, 49.04, 30.99, 29.62; HR MS (ESI): *m/z* calcd. for C_24_H_28_N_5_O_6_Na [M − H]^−^: 482.2040, found: 482.2038; Elemental analysis: calcd. for C_24_H_29_N_5_O_6_: C, 59.62; H, 6.05; N, 14.48; found: C, 59.28; H, 6.07; N, 14.21.

### 3.3. Binding Studies

#### 3.3.1. Typical Procedure for ^1^H NMR Titrations

All of the reagents were weighted separately on an analytical balance (readability to 0.01 mg) in screw-capped vials sealed with Teflon-covered septa. A DMSO-d_6_ + 0.5% H_2_O (*w*/*w*) mixture was prepared and used as the solvent. All the solvents/solutions manipulations were performed using gas-tight Hamilton glass syringes. Titrant was prepared by dissolving TBACl in a solution of the receptor to avoid dilution of the receptor during the titration. To a solution of host (600 μL, 0.01 M) in a screw-capped NMR tube sealed with Teflon-covered septum seal, appropriate aliquots of titrant (up to 10 equiv., 0.3 M, dissolved in a host solution to avoid dilution) were added with a 25 μL gas-tight microsyringe. An ^1^H NMR spectrum was recorded after each addition of the salt, and the temperature inside the NMR probe was held constant at 25 °C. Association constants were calculated based on the changes in the chemical shifts of the most affected protons in the ligands, as indicated in each case below.

#### 3.3.2. Typical Procedure for UV-Vis Titrations

All of the reagents were weighted separately on an analytical balance (readability to 0.01 mg) in screw-capped vials sealed with Teflon-covered septa. A DMSO-d_6_ + 0.5% H_2_O (*w*/*w*) mixture was prepared and used as the solvent. All the solvents/solutions manipulations were performed using gas-tight Hamilton glass syringes. Titrants were prepared by dissolving an appropriate amount of TBA salt in the solution of the receptor, in order to avoid the dilution of the receptor during the titration. To a solution of host (3 mL, 10^−4^ M) in a septum-sealed screw-caped precision cell made of SUPRASIL Quartz (light path = 10 mm), appropriate aliquots of titrant (7.5 × 10^−3^ M, dissolved in host solution to avoid dilution) were added with a 25 μL gas-tight microsyringe. The absorption spectra were recorded between 280 and 600 nm after each addition of the salt at 25 °C. Association constants were calculated based on the changes in absorbance at fixed wavelengths, as detailed below.

#### 3.3.3. Data Fitting

The association constants were determined based on the NH_amide_ and NH_carb._ resonances using global fitting analysis performed using the BindFit web applet and the 1:1 binding model (Nelder-Mead method, ‘Subtract initial values’ = ON). In all cases, the 1:1 model afforded very good fits to the experimental data and reasonable binding constants.

The UV-vis titration data were fitted with HypSpec software. The association constants and molar absorption coefficients of the complexes were set as the free parameters for fitting. The logarithms of the association constants were averaged (arithmetic mean calculated from at least two separate experiments).

### 3.4. Self-Dissociation Studies

In a typical procedure for trifluoromethanesulfonic acid titration, all of the reagents were weighted separately on an analytical balance (readability to 0.01 mg) in screw-capped vials sealed with Teflon-covered septa. A DMSO-d_6_ + 0.5% H_2_O (*w/w*) mixture was prepared and used as the solvent. All the solvents/solutions manipulations were performed using gas-tight Hamilton glass syringes. Titrant was prepared by dissolving TfOH in a solution of the receptor to avoid dilution of the receptor during the titration.

To a solution of host (3 mL, 10^−4^ M) in a septum-sealed screw-cap precision cell made of SUPRASIL Quartz (light path = 10 mm), appropriate aliquots of titrant (7.5 × 10^−3^ M, dissolved in host solution to avoid dilution) were added with a 25 μL gas-tight microsyringe. The absorption spectra were recorded between 280 and 600 nm after each addition of the acid at 25 °C.

### 3.5. Single Crystal X-ray Diffraction Analysis

Good quality single crystals for X-ray structural investigations were obtained by slow diffusion of pentane into dichloroethane solutions containing receptors **2**, **3** or **4** and Ph_4_PCl. Diffraction data were collected using an Agilent Technologies SuperNova Dual Source diffractometer with the CuKα radiation (λ = 1.54184 Å) for **2** and MoKα radiation (λ = 0.71073 Å) for **3** and **4**. The lattice parameters were determined based on a least-squares fit of the optimized setting angles of the reflections collected by using the CrysAlis CCD software [33]. Data were reduced using the CrysAlis RED program [33]. The Gaussian numerical absorption correction was applied using a multifaceted crystal model implemented in the SCALE3 ABSPACK scaling algorithm [33]. The structures were solved using Olex2 [34] with the ShelXT [35] structure solution program using Intrinsic Phasing and refined with the ShelXL [36] refinement package with least squares minimization. All H-atoms were positioned geometrically. The crystallographic data are summarized in Table S1, Supplementary Materials. The values of bond lengths and valence angles are provided in Tables S2−S3, Supplementary Materials.

## 4. Conclusions

In this article, we described the synthesis of two novel building blocks for the construction of anion receptors: 1,8-diamino-3,6-dicyanocarbazole (**12**) and 1,8-diamino-3,6-dinitrocarbazole (**16**). Both of these compounds are interesting not only in the context of anion recognition, but also as high-potential synthons for organic synthesis in general, because they comprise as many as five amino group equivalents on a single carbazole core.

We also synthesized two new 1,8-diamidocarbazole receptors containing strongly electron-withdrawing substituents (3,6-dicyano and 3,6-dinitro). Both of these molecules exhibited significantly enhanced anion affinities relative to the unsubstituted and 3,6-dichloro substituted receptors, but they were also much more prone to proton abstraction. Carboxylate, phosphate, and sulfate anions significantly deprotonated these receptors in wet DMSO, which limited their utility for anion recognition. Thus, we experimentally established the limits of anion affinity enhancement that could be achieved by the substitution of the carbazole platform.

The new 3,6-dinitro substituted receptor **4** acted as colorimetric anion sensor in wet DMSO, exhibiting distinct color changes upon interacting with basic anions. Unfortunately, however, these changes were mostly caused by receptor deprotonation, and therefore lacked specificity. In contrast to **4**, the 3,6-dicyano-substituted receptor **3** was colorless and produced no significant color changes upon addition of anions. However, it also underwent deprotonation in the presence of various basic anions, which manifested under UV irradiation as marked enhancements in fluorescence intensity.

Owing to their very high hydrogen bond donating abilities, combined with their relatively high Brønsted acidities, diamidocarbazoles similar to **3** and **4** may be applicable in organocatalysis [37] or pH-switchable anion transport through lipophilic membranes [38,39,40,41]. Research in these directions is ongoing in our laboratories and will be published in due course [42].

## Figures and Tables

**Table 1:** Hydrogen bonding distances and selected torsion angles in the X-ray crystal structures of chloride complexes of **2**, **3** and **4**.

Parameter	Atom	2 × Cl^−^	3 × Cl^−^	4 × Cl^−^
Bond length, Å	Cl^−^∙∙∙NH_carb._	2.223	2.198	2.170
	Cl^−^∙∙∙NH_amide1_	2.567	2.563	2.533
	Cl^−^∙∙∙NH_amide2_	2.620	2.569	2.595
	Cl^−^∙∙∙CH_t-Bu_	2.850	2.910	2.878
	Cl^−^∙∙∙Ph_4_P^+^	2.771	2.835	2.778
Torsion angle φ, ˚	C2-C1-N_amide1_-C_amide1_	6.83	3.80	5.00
	C7-C8-N_amide2_-C_amide2_	0.23	−5.22	0.38
Torsion angle φ, ˚	C9a-C1-N_amide1_-C_amide1_	−175.26	−178.32	−176.94
	C8a-C8-N_amide2_-C_amide2_	−178.76	177.43	174.60

**Table 2:** Binding constants of receptors **1**-**4** with various anions in DMSO-d_6_ + 0.5% H_2_O at 298K.

Receptor	*K_a_* Cl^−^	*K_a_* PhCOO^−^	*K_a_* H_2_PO_4_^−^	*K_a_* CH_3_COO^−^
**1**	48 **^[a]^**	10^3.67 **[a]**^	10^4.01 **[a]**^	10^4.07 **[a]**^
**2**	159 **^[a]^**	10^4.47 **[a]**^	10^4.91 **[a]**^	10^4.95 **[a]**^
**3**	309	deprotonation	10^5.4^ *	n.d.
**4**	347	deprotonation	deprotonation	n.d.

^[a]^ Value from ref. [22];. n.d. = not deterimined; * estimated complexation constant, disregarding partial deprotonation.

## Data Availability

Crystallographic data for the structures in this paper have been deposited with the Cambridge Crystallographic Data Centre as supplementary publication number CCDC 2074344 and 2074345. Copies of the data can be obtained, free of charge, on application to CCDC [e-mail: deposit@ccdc.cam.ac.uk, website: www.ccdc.cam.ac.uk].

## Supplementary Materials

The following are available online: Figure S1: ^1^H NMR spectrum of 3,6-dicyjano-1,8-dinitrocarbazole in DMSO-d_6_; Figure S2: ^13^C NMR spectrum of 3,6-dicyjano-1,8-dinitrocarbazole in DMSO-d_6_; Figure S3: Mass spectrum of 3,6-dicyjano-1,8-dinitrocarbazole; Figure S4: ^1^H NMR spectrum of 1,8-diamino-3,6-dicyjanocarbazole in DMSO-d_6_; Figure S5: ^13^C NMR spectrum of 1,8-diamino-3,6-dicyjanocarbazole in DMSO-d_6_; Figure S6: Mass spectrum of 1,8-diamino-3,6-dicyjanocarbazole; Figure S7: ^1^H NMR spectrum of **3** in DMSO-d_6_; Figure S8: ^13^C NMR spectrum of **3** in DMSO-d_6_; Figure S9: Mass spectrum of **3**; Figure S10: ^1^H NMR spectrum of 1,8-diamino-3,6-dinitrocarbazole in DMSO-d_6_; Figure S11: ^13^C NMR spectrum of 1,8-diamino-3,6-dinitrocarbazole in DMSO-d_6_; Figure S12: Mass spectrum of 1,8-diamino-3,6-dinitrocarbazole; Figure S13: ^1^H NMR spectrum of **4** in DMSO-d_6_; Figure S14: ^13^C NMR spectrum of **4** in DMSO-d_6_; Figure S15: ^1^H ROESY spectrum of **4** in DMSO-d_6_; Scheme S1: Amide groups conformations in **4**; Figure S16: Mass spectrum of **4**; ^1^H NMR spectra of titration with TBACl in DMSO-d_6_ + 0.5% H_2_O, raw data, titration curves obtained from chemical shifts of NH_carb._ and NH_amide_ protons; UV-vis spectra from titrations with TBA salts in DMSO-d_6_ + 0.5% H_2_O, raw data; titration curve, UV-vis spectra of self-dissociation reversing titration with TfOH in DMSO-d_6_ + 0.5%H_2_O, titration curves; crystallographic data and refinement details.
