# Supplementary material for: Carbazole-Based Colorimetric Anion Sensors
Source: Molecules. 2021 May 27;26(11):3205. doi: 10.3390/molecules26113205 (PMC8199442; doi:10.3390/molecules26113205)

Supporting Information

Carbazole-based colorimetric anion sensors

Krystyna Maslowska-Jarzyna, Maria L. Korczak, Jakub Wagner and Michał J. Chmielewski*
Faculty of Chemistry, Biological and Chemical Research Centre, University of Warsaw
Żwirki i Wigury 101, 02-089 Warszawa, Poland

E-mail: mchmielewski@chem.uw.edu.pl

Contents

1 NMR spectra 3
2 Binding studies 9
2.1 Anion binding studies of receptor **3** 9
2.1.1 ^{1}H NMR titration of **3** with Cl^- in DMSO/0.5%H_2O 9
2.1.2 UV-Vis titration of **3** with $H_2PO_4^-$ in DMSO/0.5%H_2O 11
2.1.3 UV-Vis titration of **3** with $PhCOO^-$ in DMSO/0.5%H_2O 13
2.1.4 UV-Vis titration of **3** with OH^- in DMSO/0.5%H_2O 14
2.2 Anion binding studies of receptor **4** 15
2.2.1 ^{1}H NMR titration of **4** with Cl^- in DMSO/0.5%H_2O 15
2.2.2 UV-Vis titration of **4** with $H_2PO_4^-$ in DMSO/0.5%H_2O 17
2.2.3 UV-Vis titration of **4** with $PhCOO^-$ in DMSO/0.5%H_2O 18
2.2.4 UV-Vis titration of **4** with Cl^- in DMSO/0.5%H_2O 19
2.2.5 UV-Vis titration of **4** with OH^- in DMSO/0.5%H_2O 20
3 Self-dissociation studies 21
3.1 Self-dissociation studies of receptor **3** 21
3.2 Self-dissociation studies of receptor **4** 22
4 Crystallographic data and refinement details 23
4.1 ^{1}H NMR of crystallised complex [**4** $\times$ Ph_4PCl] dissolved in $CDCl_3$ 28

1 NMR spectra

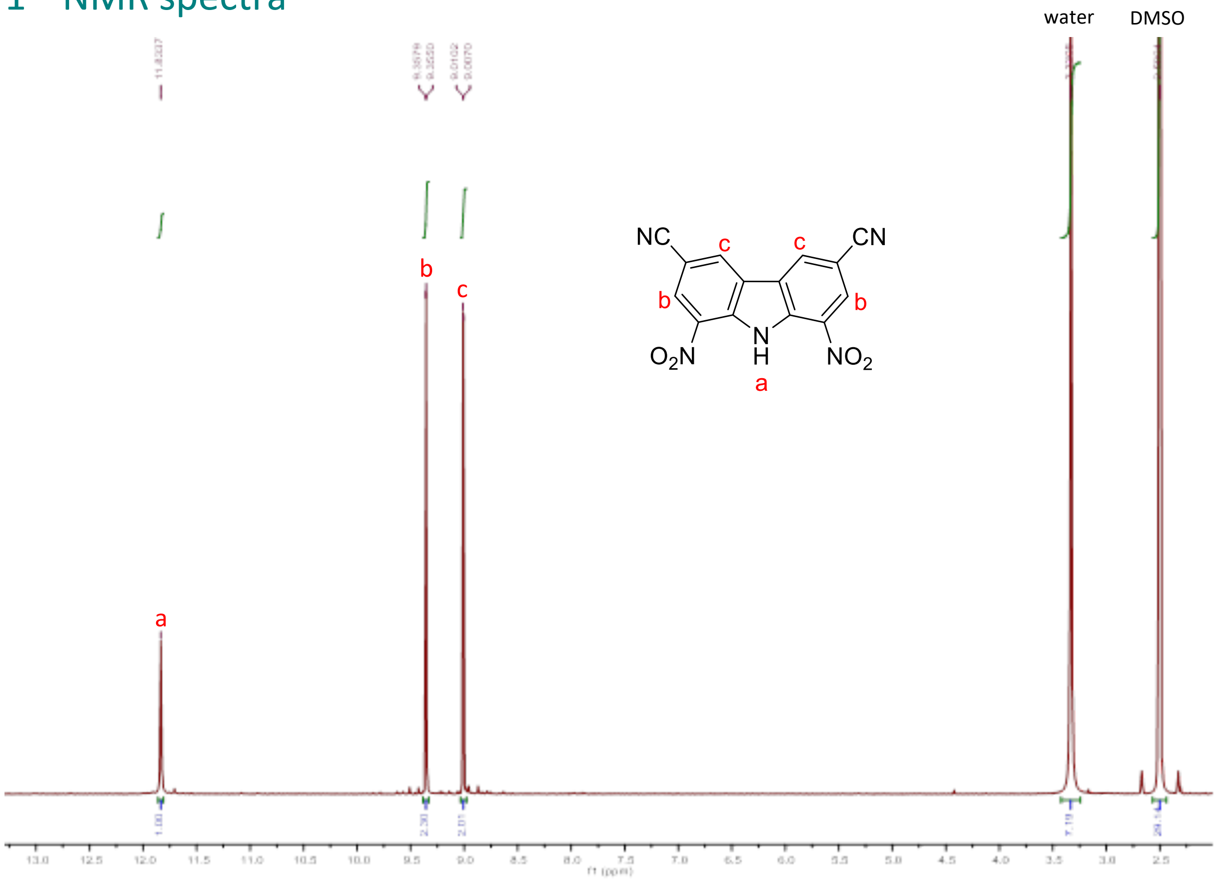

Figure S1. ^{1}H NMR spectrum of 3,6-dicyjano-1,8-dinitrocarbazole in DMSO-d_6.

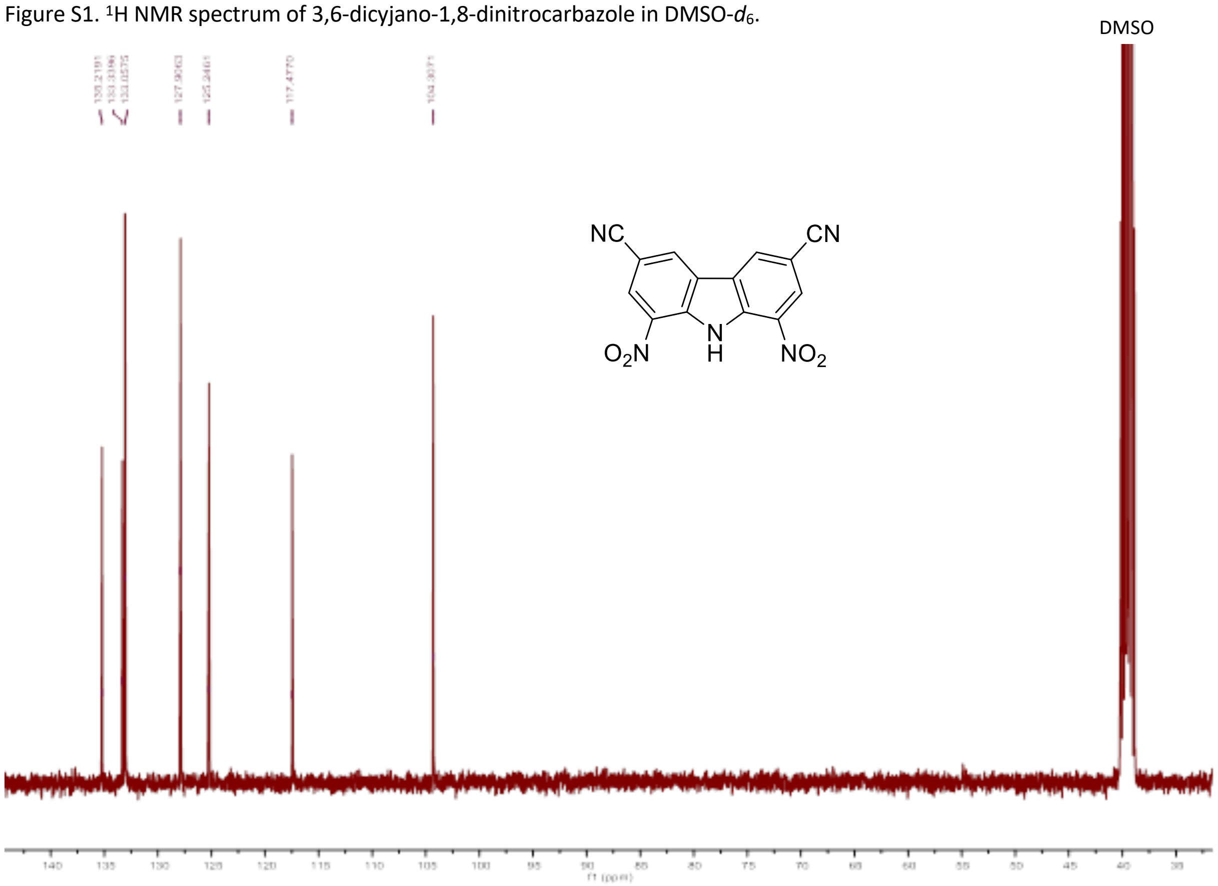

Figure S2. ^{13}C NMR spectrum of 3,6-dicyjano-1,8-dinitrocarbazole in DMSO-d_6.

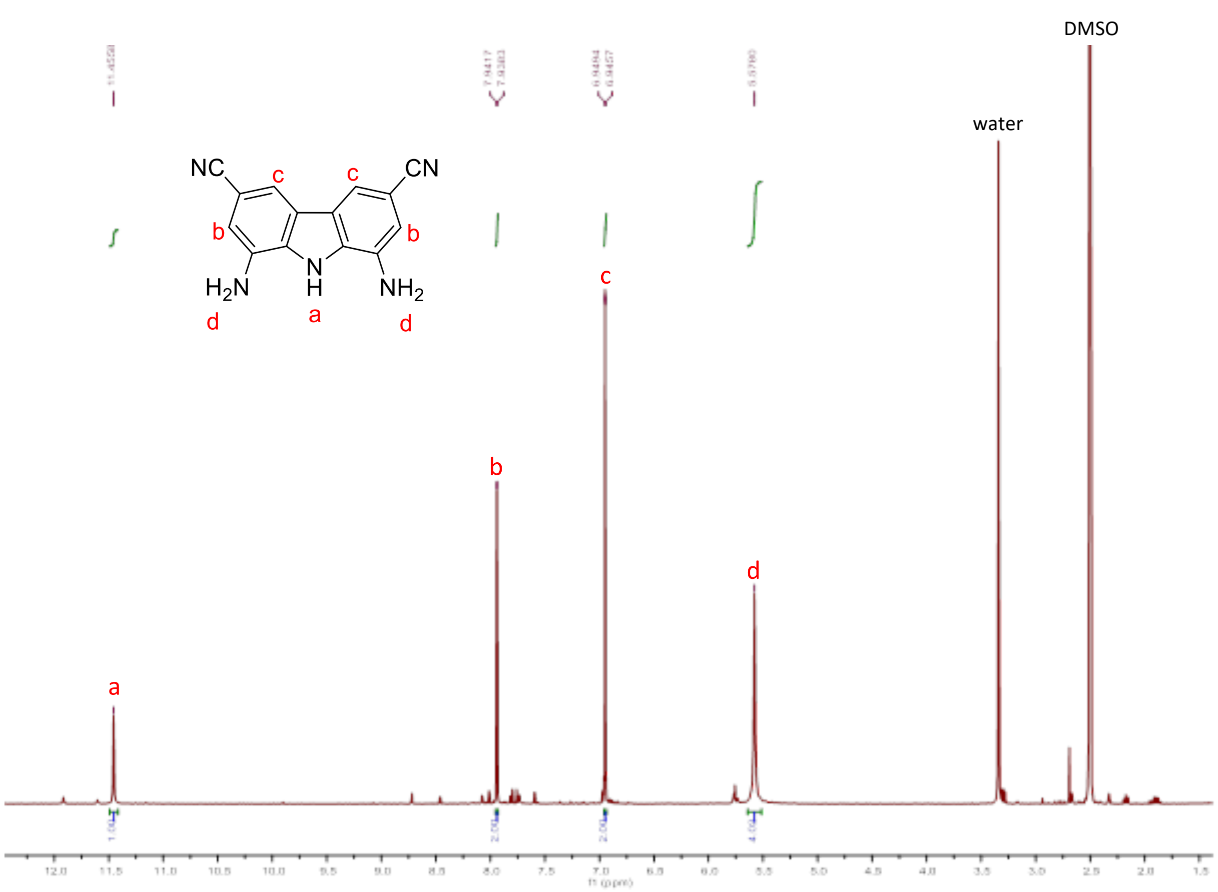

Figure S3. ^{1}H NMR spectrum of 1,8-diamino-3,6-dicyjanocarbazole in DMSO-d_6.

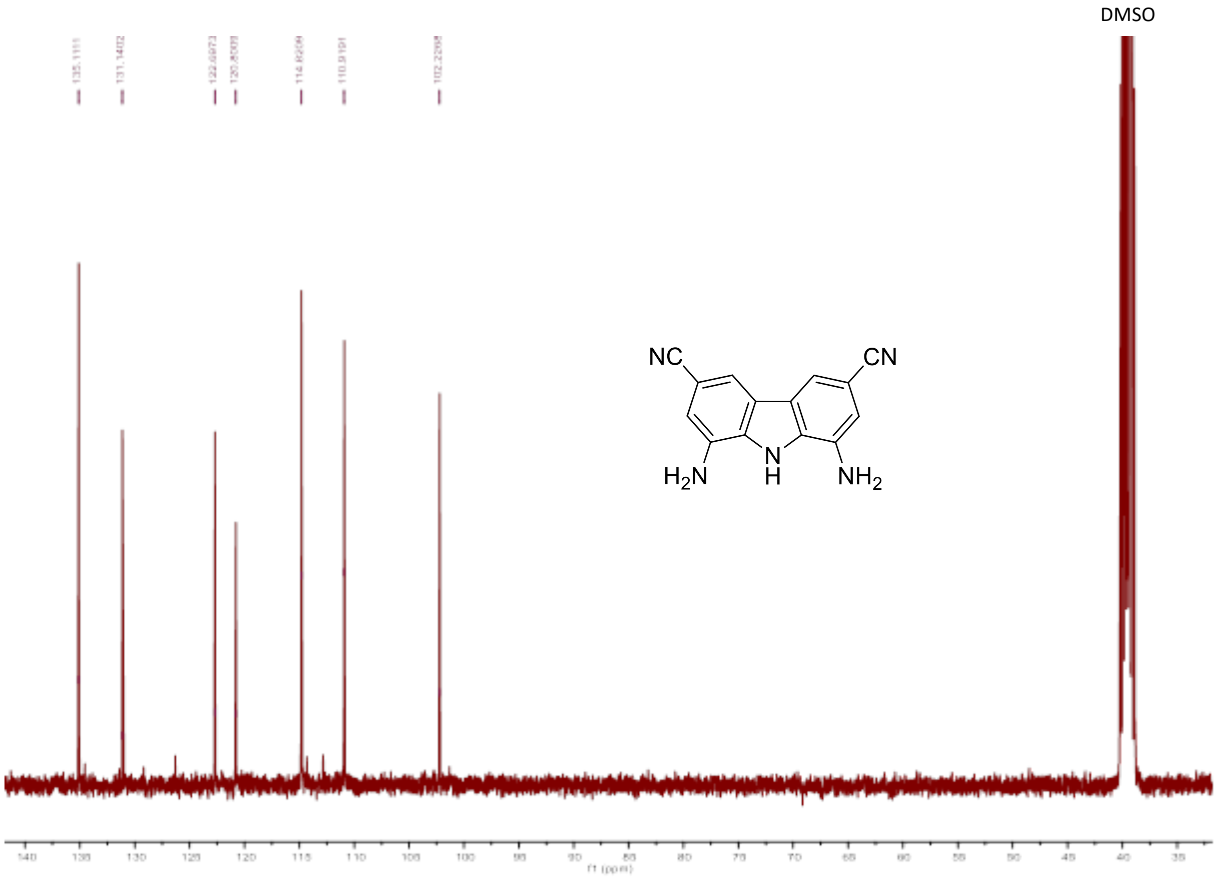

Figure S4. ^{13}C NMR spectrum of 1,8-diamino-3,6-dicyjanocarbazole in DMSO-d_6.

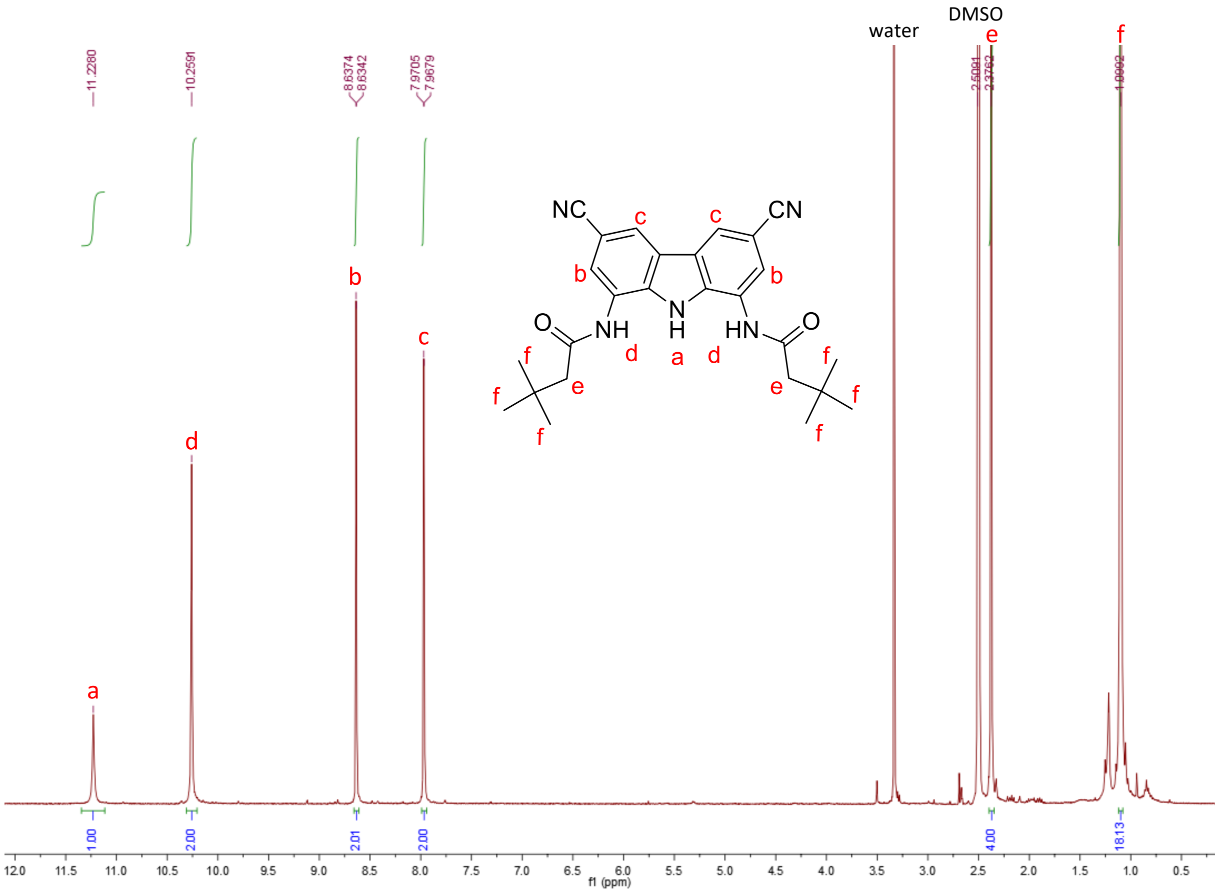

Figure S5. ^{1}H NMR spectrum of **3** in DMSO-d_6.

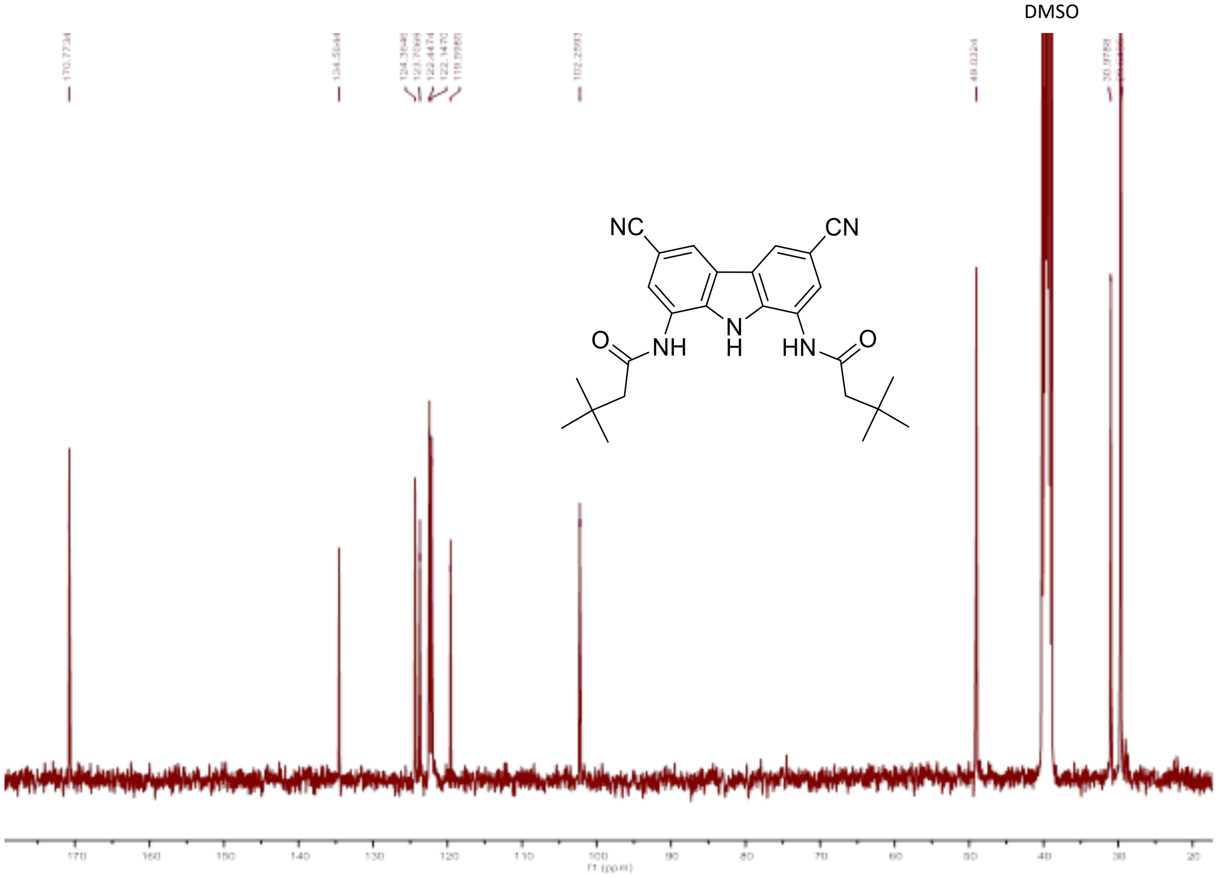

Figure S6. ^{13}C NMR spectrum of **3** in DMSO-d_6.

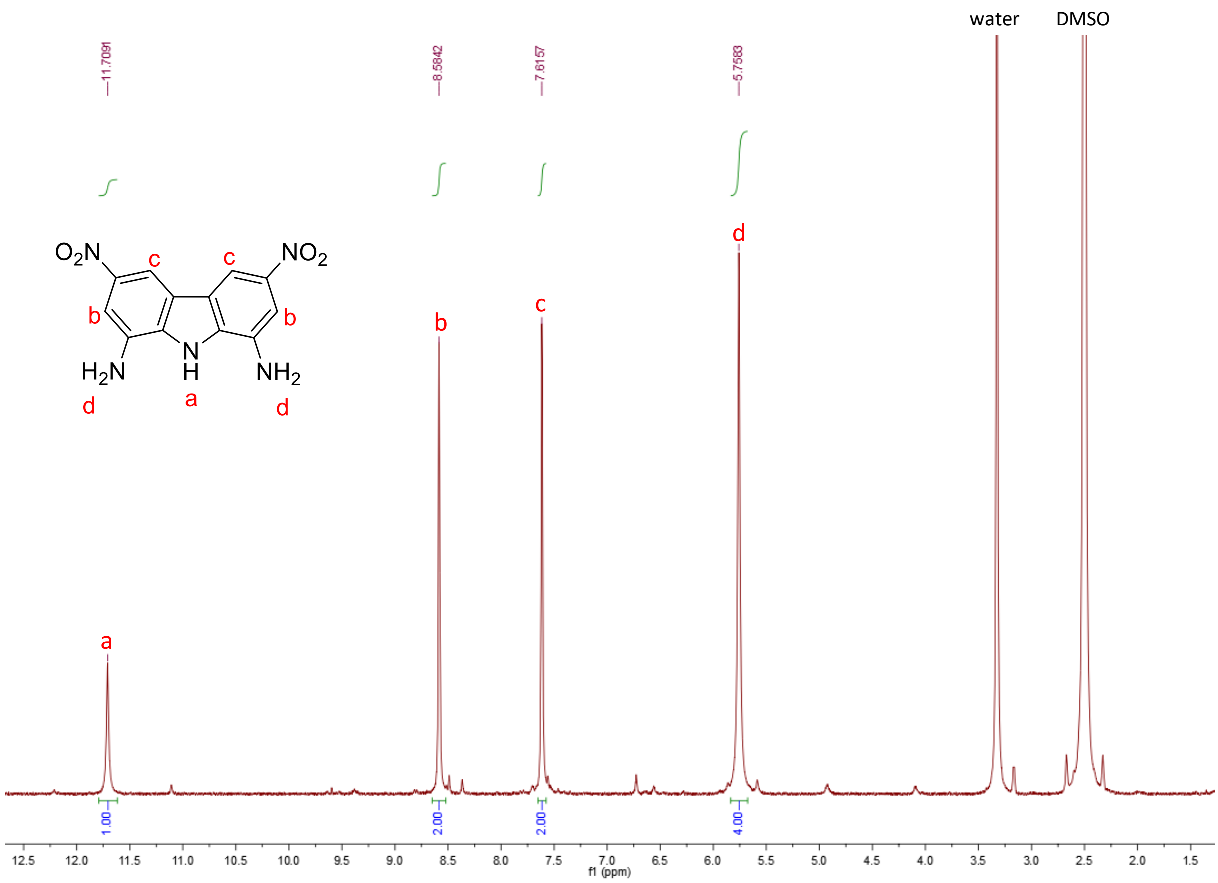

Figure S7. ^{1}H NMR spectrum of 1,8-diamino-3,6-dinitrocarbazole in DMSO-d_6.

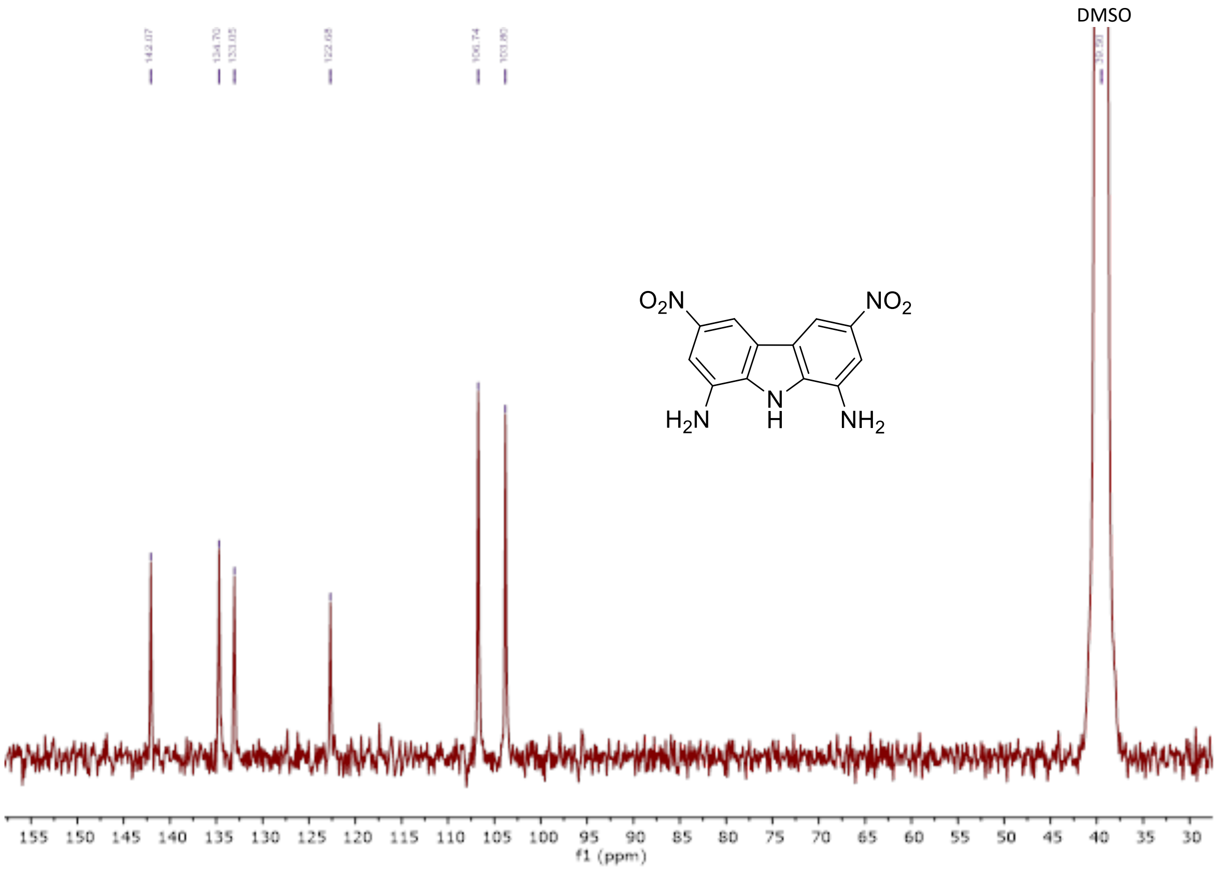

Figure S8. ^{13}C NMR spectrum of 1,8-diamino-3,6-dinitrocarbazole in DMSO-d_6.

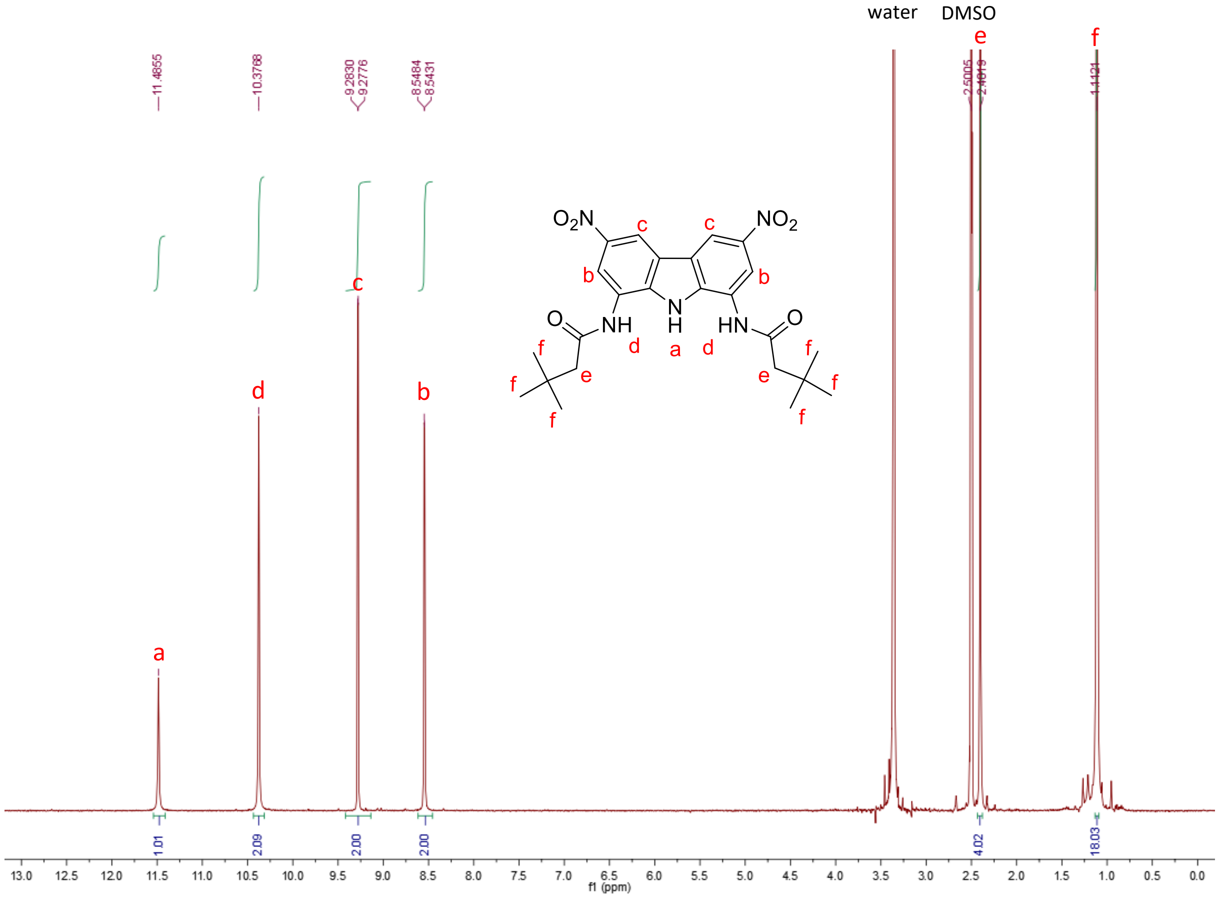

Figure S9. ^{1}H NMR spectrum of **4** in DMSO-d_6.

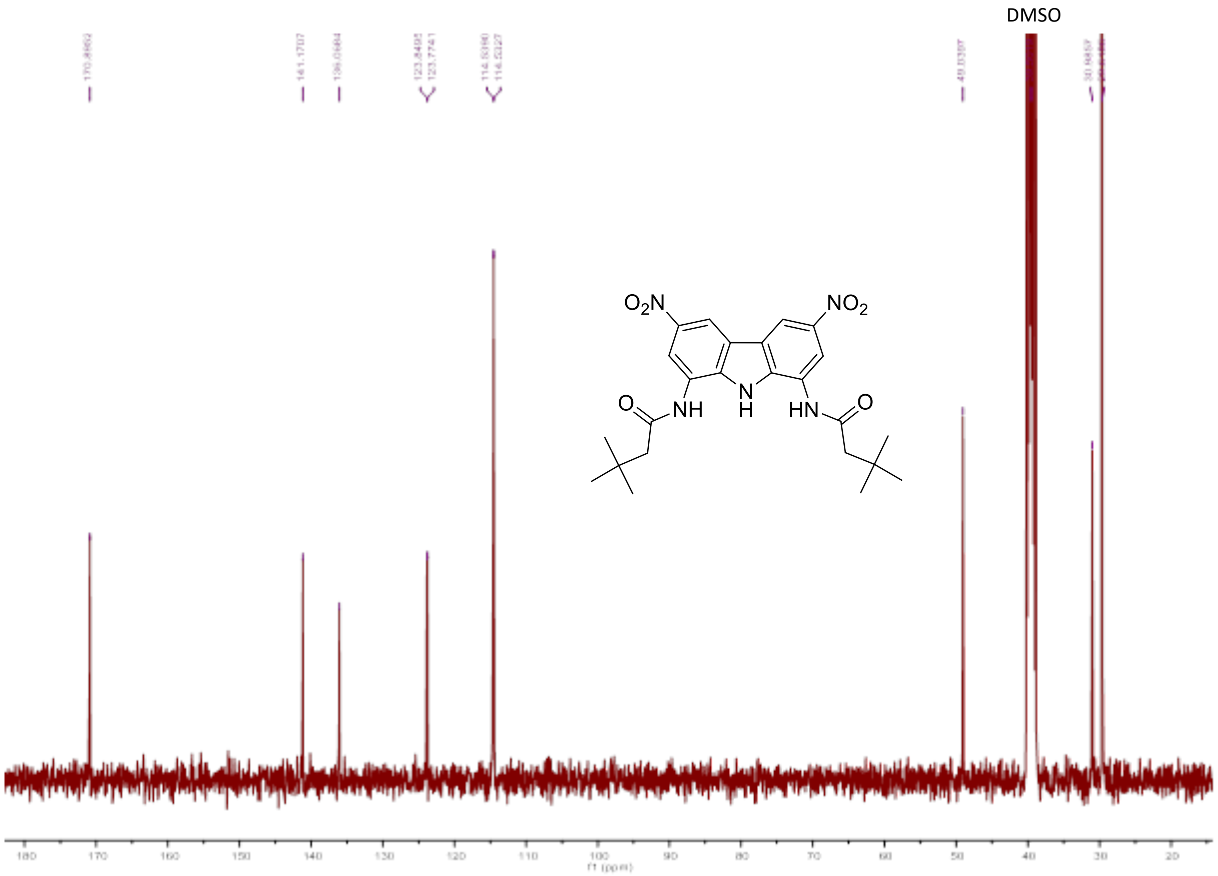

Figure S10. ^{13}C NMR spectrum of **4** in DMSO-d_6.

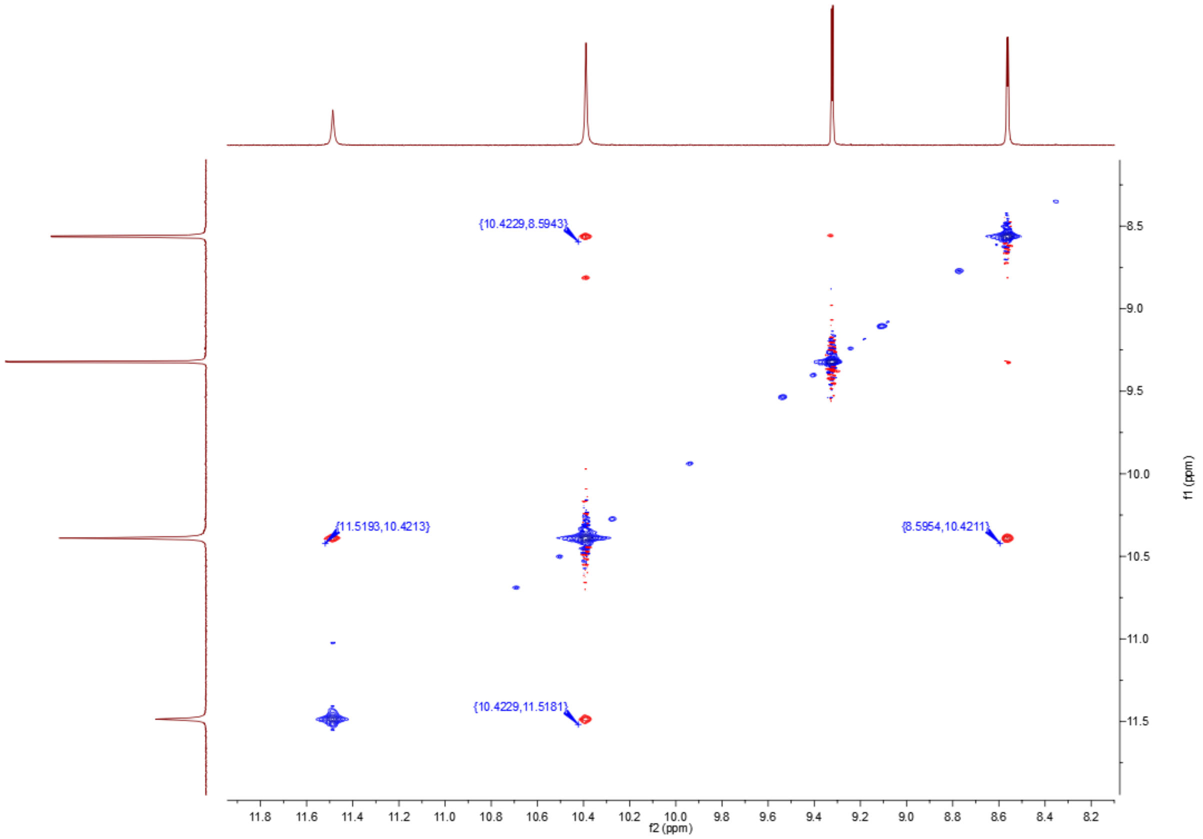

Figure S11. ^{1}H ROESY spectrum of **4** in DMSO-d_6.

2D ROESY (*Rotation Frame Nuclear Overhauser Effect Spectroscopy*) spectrum reveals which hydrogen atoms of **4** are close to each other in space. The protons of the amide groups interact with both carbazole proton NH at 11.52 ppm and CH2/7 protons at 8.60 ppm, what means that both *syn* and *anti*-conformations of the amide groups are populated in DMSO solution, as shown on Scheme S1.

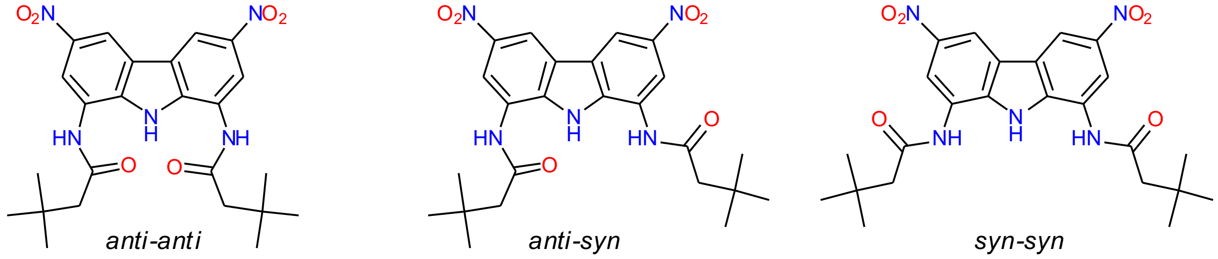

Scheme S1. Amide groups conformations in **4**.

2 Binding studies

2.1 Anion binding studies of receptor **3**

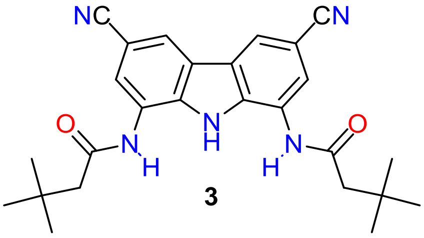

2.1.1 ^{1}H NMR titration of **3** with Cl^- in DMSO/0.5%H_2O

^{1}H NMR titration of 0.01 M solution of receptor **3** in DMSO-d_6/0.5% H_2O with 0.3 M solution of TBACl (dissolved in the solution of receptor **3**).

a) ^{1}H NMR spectra

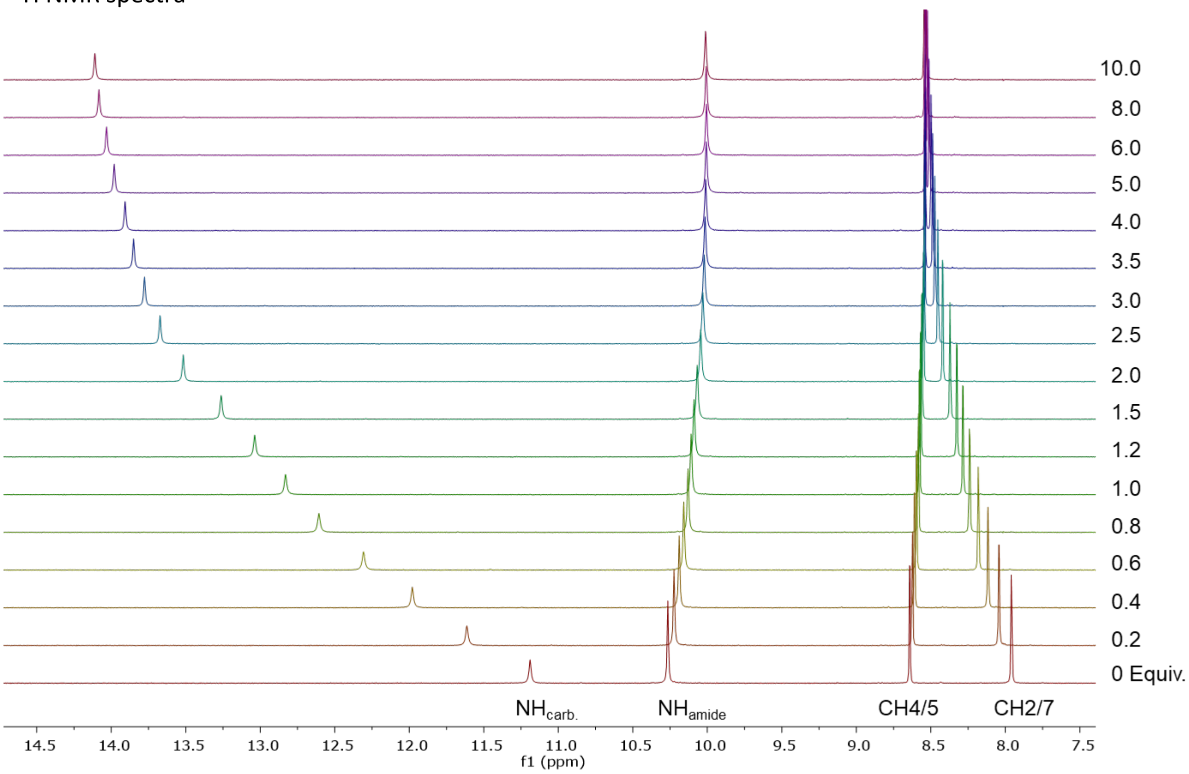

b) Raw data

Added volume of titrant [µL]	Equivalents of TBACl	Chemical shift [ppm]	
		$NH_{carb.}$	NH_{amide}
0.00	0	11.4867	10.3867
4.00	0.2	11.7219	10.3584
8.00	0.4	11.9426	10.3328
16.25	0.6	12.3577	10.2793
25.00	0.8	12.7551	10.2315
33.75	1.0	13.0694	10.1919
42.75	1.2	13.3287	10.1604
52.25	1.5	13.5376	10.1350
66.75	2.0	13.7653	10.1074
91.75	2.5	13.9954	10.0795
120.00	3.0	14.1334	10.0631
150.00	3.5	14.2170	10.0537
182.50	4.0	14.2728	10.0477
218.25	5.0	14.3128	10.0438
300.00	6.0	14.3676	10.0416
400.00	8.0	14.3982	10.0387
686.00	9.0	14.4319	10.0397
911.00	10.0	14.4402	10.0409

c) Titration curves of $NH_{carb.}$ and NH_{amide} protons

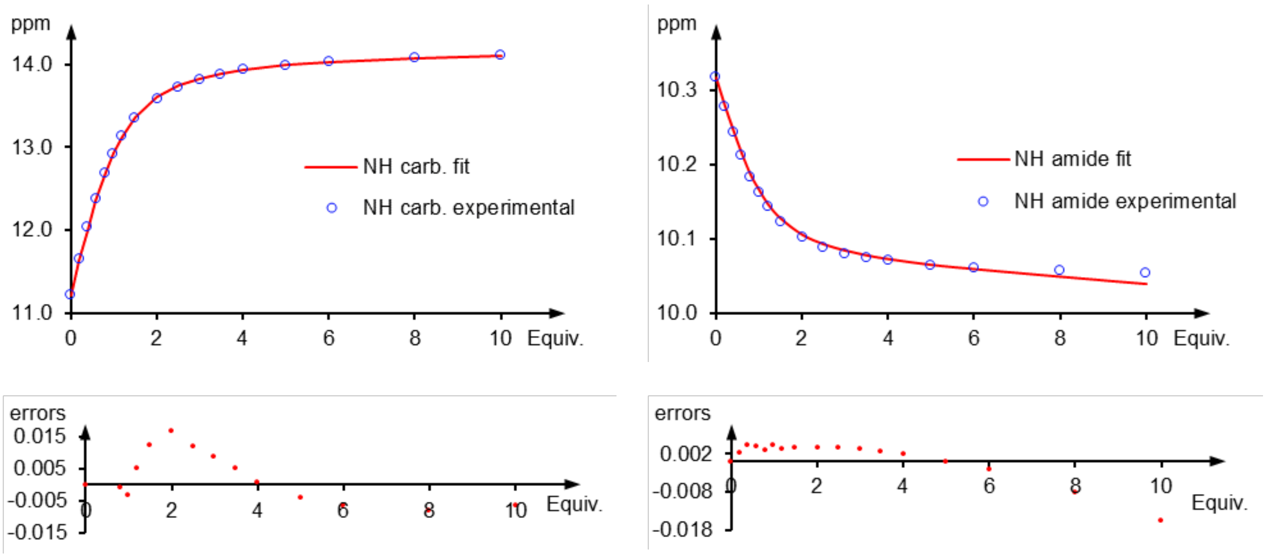

d) Logarithm of the binding constant log K derived from simultaneous fitting of 1:1 model to two selected protons using Bindfit:

log K: 2.497

e) Logarithm of binding constant log K derived from the experiment repeated according to the same methodology:

log K: 2.491

f) Logarithm of binding constant log K averaged from the two experiments:

log K: 2.494

2.1.2 UV-Vis titration of **3** with $H_2PO_4^-$ in DMSO/0.5%H_2O

UV-Vis titration of 1×10^{-4} M solution of receptor **3** in DMSO/0.5% H_2O with 0.0075 M solution of $TBAH_2PO_4$ (dissolved in the solution of receptor **3**).

a) UV-Vis spectra

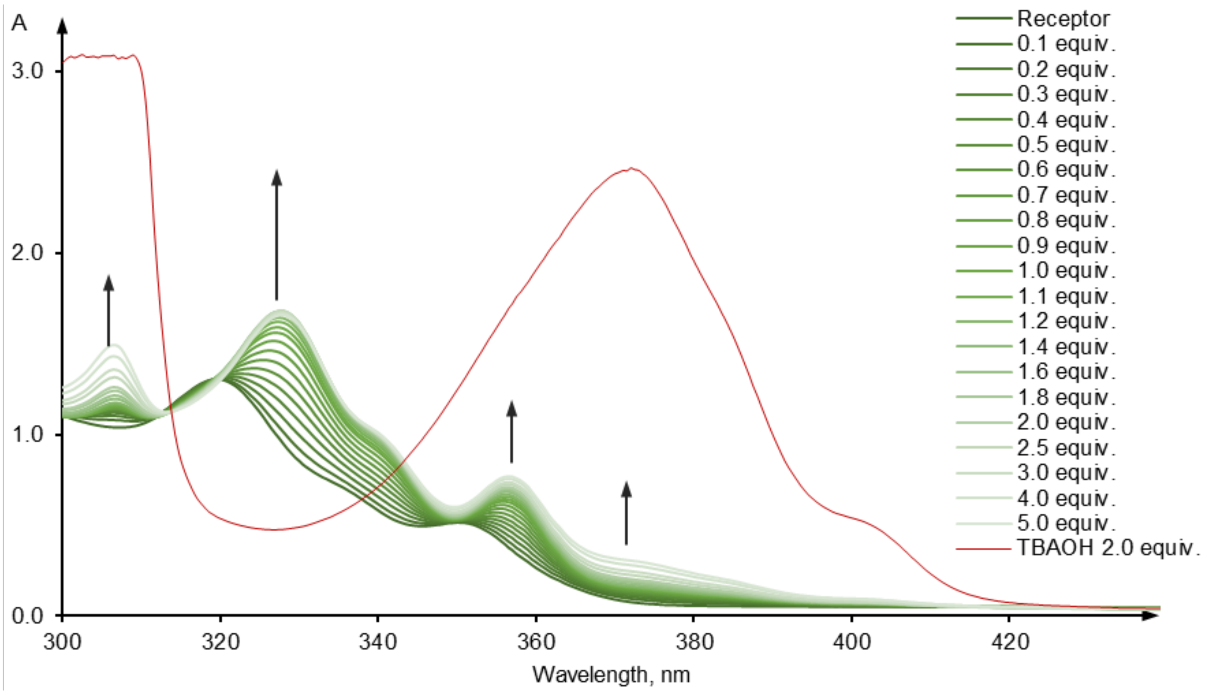

b) Raw data

Equivalents of $TBAH_2PO_4$	307 nm	328 nm	337.5 nm	357 nm	372 nm
0.0	1.038	0.948	0.655	0.361	0.075
0.1	1.080	1.015	0.693	0.398	0.098
0.2	1.101	1.088	0.733	0.431	0.109
0.3	1.117	1.163	0.772	0.460	0.117
0.4	1.128	1.238	0.813	0.491	0.124
0.5	1.137	1.309	0.852	0.520	0.131
0.6	1.143	1.377	0.890	0.548	0.136
0.7	1.147	1.446	0.926	0.576	0.141
0.8	1.149	1.506	0.960	0.600	0.145
0.9	1.155	1.554	0.988	0.621	0.150
1.0	1.159	1.589	1.007	0.637	0.154
1.1	1.166	1.617	1.025	0.651	0.159
1.2	1.175	1.642	1.040	0.664	0.165
1.4	1.195	1.663	1.054	0.679	0.175
1.6	1.218	1.677	1.064	0.693	0.187
1.8	1.242	1.682	1.070	0.702	0.197
2.0	1.262	1.683	1.073	0.709	0.206
2.5	1.314	1.681	1.077	0.724	0.229
3.0	1.358	1.677	1.080	0.737	0.250
4.0	1.430	1.670	1.082	0.754	0.279
5.0	1.491	1.661	1.083	0.768	0.305

c) Titration curves at 337.5 nm.

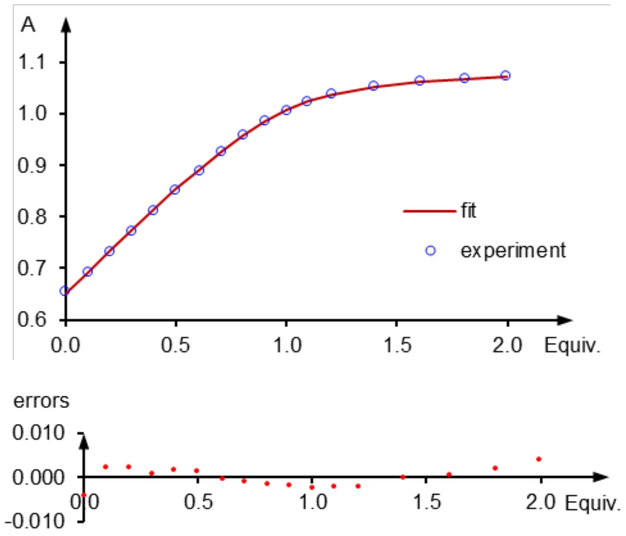

d) Logarithm of the binding constant log K derived from simultaneous fitting of 1:1 model to three selected wavelengths using HypSpec:

log K: 5.370

2.1.3 UV-Vis titration of **3** with $PhCOO^-$ in DMSO/0.5%H_2O

UV-Vis titration of 1×10^{-4} M solution of receptor **3** in DMSO/0.5% H_2O with 0.0075 M solution of TBAPhCOO (dissolved in the solution of receptor **3**).

a) UV-Vis spectra

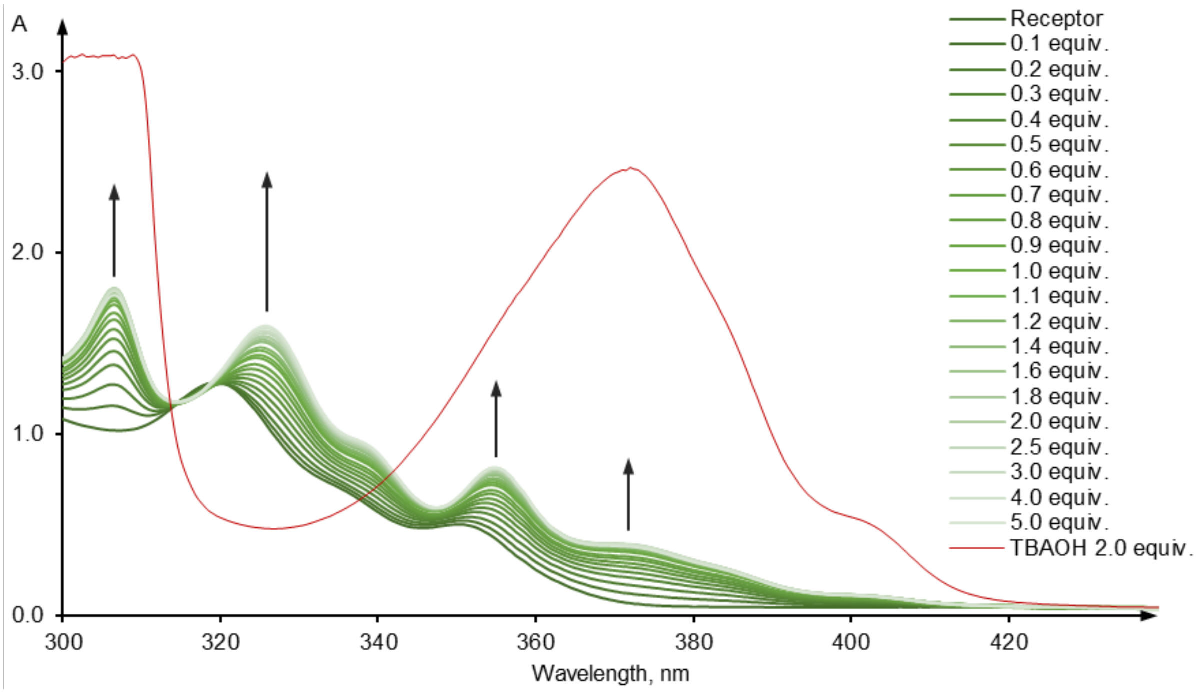

b) Raw data

Equivalents of TBAPhCOO	307 nm	326 nm	357 nm	372 nm
0.0	1.022	1.046	0.349	0.065
0.1	1.154	1.074	0.393	0.119
0.2	1.274	1.113	0.438	0.167
0.3	1.376	1.158	0.480	0.207
0.4	1.453	1.204	0.518	0.239
0.5	1.520	1.248	0.552	0.265
0.6	1.573	1.289	0.581	0.288
0.7	1.627	1.331	0.611	0.310
0.8	1.661	1.363	0.633	0.325
0.9	1.706	1.404	0.662	0.349
1.0	1.733	1.426	0.677	0.359
1.1	1.743	1.450	0.689	0.365
1.2	1.758	1.471	0.704	0.372
1.4	1.772	1.502	0.718	0.379
1.6	1.786	1.521	0.733	0.385
1.8	1.795	1.534	0.741	0.390
2.0	1.798	1.537	0.739	0.389
2.5	1.798	1.558	0.752	0.391
3.0	1.791	1.570	0.756	0.388
4.0	1.782	1.586	0.759	0.386
5.0	1.763	1.599	0.760	0.379

2.1.4 UV-Vis titration of **3** with OH^- in DMSO/0.5%H_2O

UV-Vis titration of 1×10^{-4} M solution of receptor **3** in DMSO/0.5% H_2O with 0.0075 M solution of TBAOH (dissolved in the solution of receptor **3**).

a) UV-Vis spectra

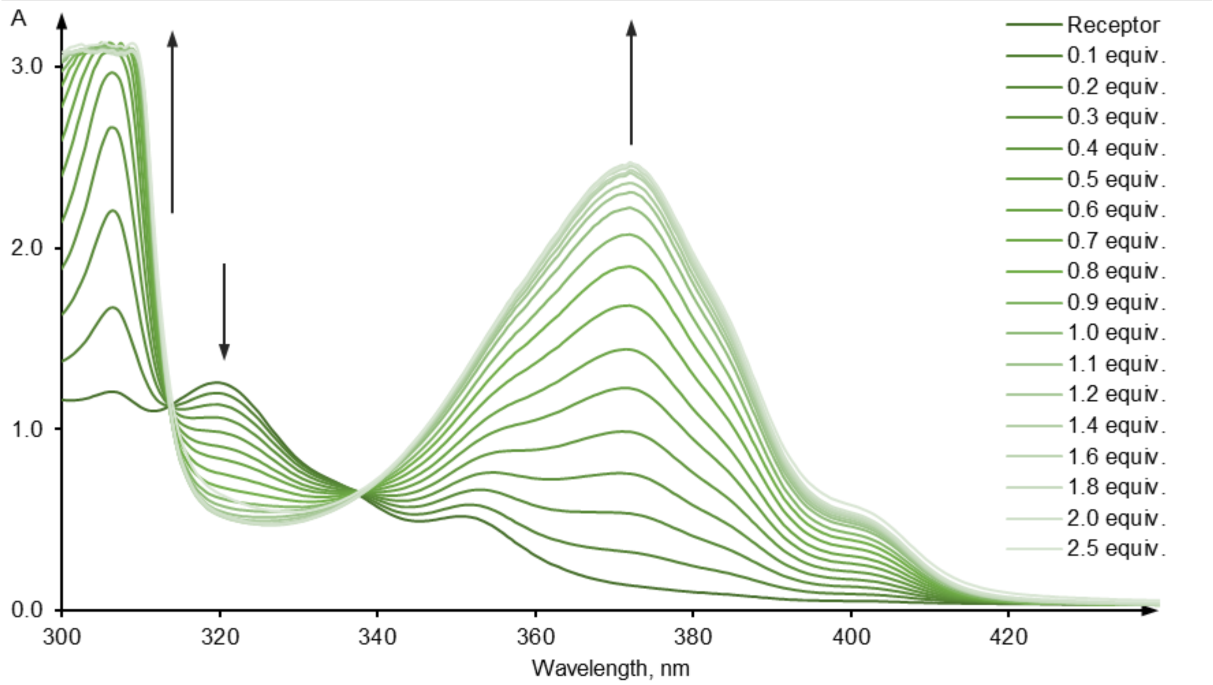

b) Raw data

Equivalents of TBAOH	307 nm	320 nm	350 nm	372 nm
0.0	1.202	1.254	0.520	0.141
0.1	1.667	1.201	0.575	0.321
0.2	2.188	1.133	0.639	0.533
0.3	2.648	1.063	0.706	0.753
0.4	2.955	0.986	0.777	0.987
0.5	3.081	0.906	0.850	1.227
0.6	3.102	0.838	0.915	1.439
0.7	3.105	0.758	0.989	1.682
0.8	3.096	0.685	1.058	1.900
0.9	3.094	0.625	1.115	2.078
1.0	3.082	0.579	1.161	2.220
1.1	3.102	0.549	1.192	2.307
1.2	3.076	0.534	1.209	2.360
1.4	3.080	0.526	1.224	2.414
1.6	3.073	0.527	1.231	2.433
1.8	3.065	0.532	1.242	2.454
2.0	3.074	0.535	1.248	2.471
2.5	3.110	0.646	1.247	2.469

2.2 Anion binding studies of receptor **4**

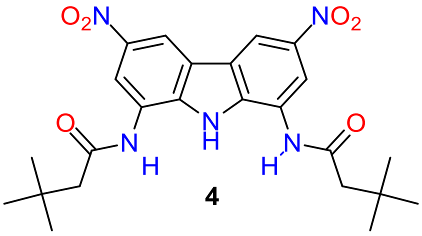

4

2.2.1 ^{1}H NMR titration of **4** with Cl^{-} in DMSO/0.5%H_2O

^{1}H NMR titration of 0.01 M solution of receptor **4** in DMSO-d_6/0.5% H_2O with 0.3 M solution of TBACl (dissolved in the solution of receptor **4**).

a) ^{1}H NMR spectra

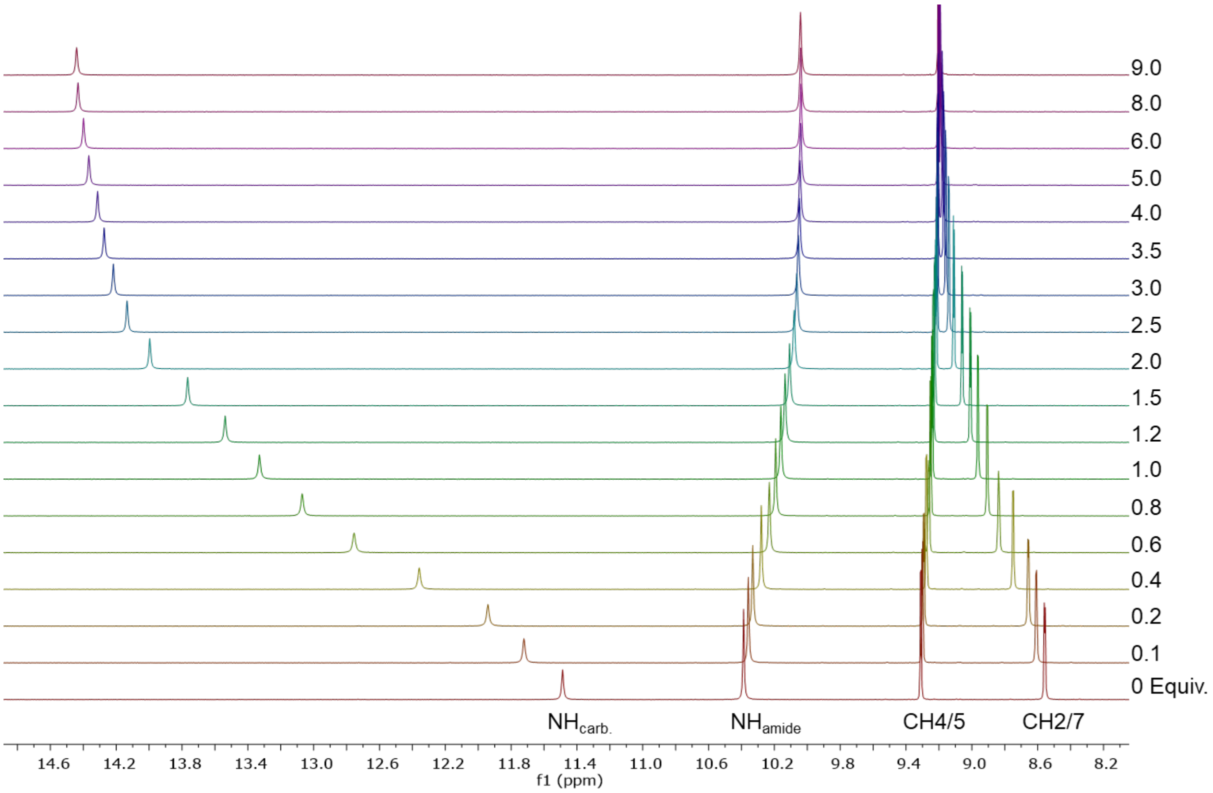

b) Raw data

Added volume of titrant [μL]	Equivalents of TBACl	Chemical shift [ppm]	
		NHcarb.	NHamide
0.00	0	11.4867	10.3867
4.00	0.1	11.7219	10.3584
8.00	0.2	11.9426	10.3328
16.25	0.4	12.3577	10.2793
25.00	0.6	12.7551	10.2315
33.75	0.8	13.0694	10.1919
42.75	1.0	13.3287	10.1604
52.25	1.2	13.5376	10.1350
66.75	1.5	13.7653	10.1074
91.75	2.0	13.9954	10.0795
120.00	2.5	14.1334	10.0631
150.00	3.0	14.2170	10.0537
182.50	3.5	14.2728	10.0477
218.25	4.0	14.3128	10.0438
300.00	5.0	14.3676	10.0416
400.00	6.0	14.3982	10.0387
686.00	8.0	14.4319	10.0397
911.00	9.0	14.4402	10.0409

c) Titration curve of $NH_{carb.}$ and NH_{amide} protons

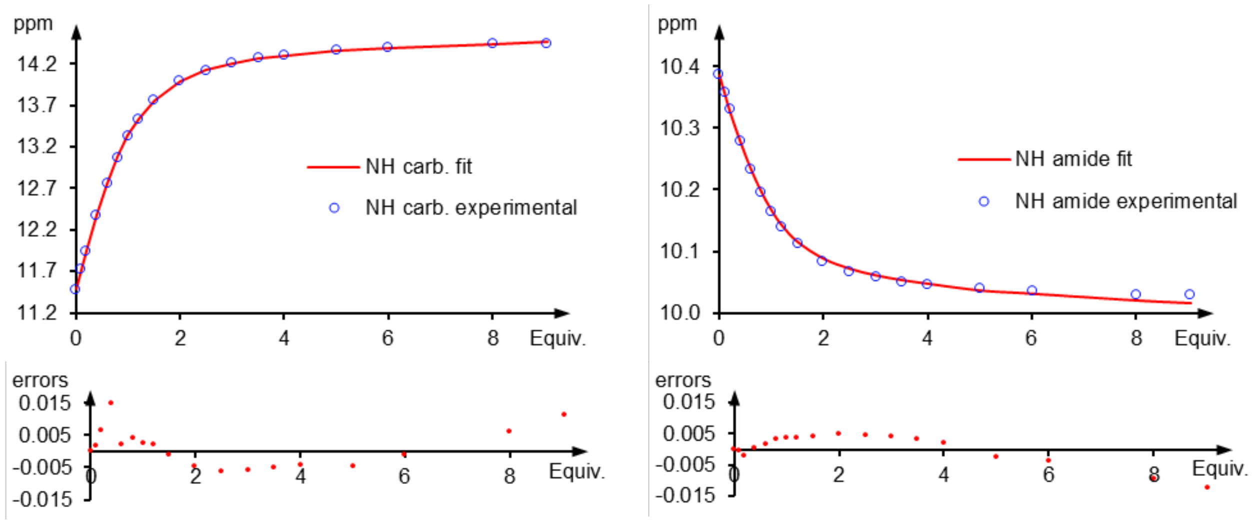

d) Logarithm of the binding constant log K derived from simultaneous fitting of 1:1 model to two selected protons using Bindfit:

log K: 2.584

e) Logarithm of the binding constant log K derived from the experiment repeated according to the same methodology:

log K: 2.491

f) Logarithm of the binding constant log K averaged from the two experiments:

log K: 2.538

2.2.2 UV-Vis titration of **4** with $H_2PO_4^-$ in DMSO/0.5%H_2O

UV-Vis titration of $1{\times}10^{-4}$ M solution of receptor **4** in DMSO/0.5% H_2O with 0.0075 M solution of $TBAH_2PO_4$ (dissolved in the solution of receptor **4**).

a) UV-Vis spectra

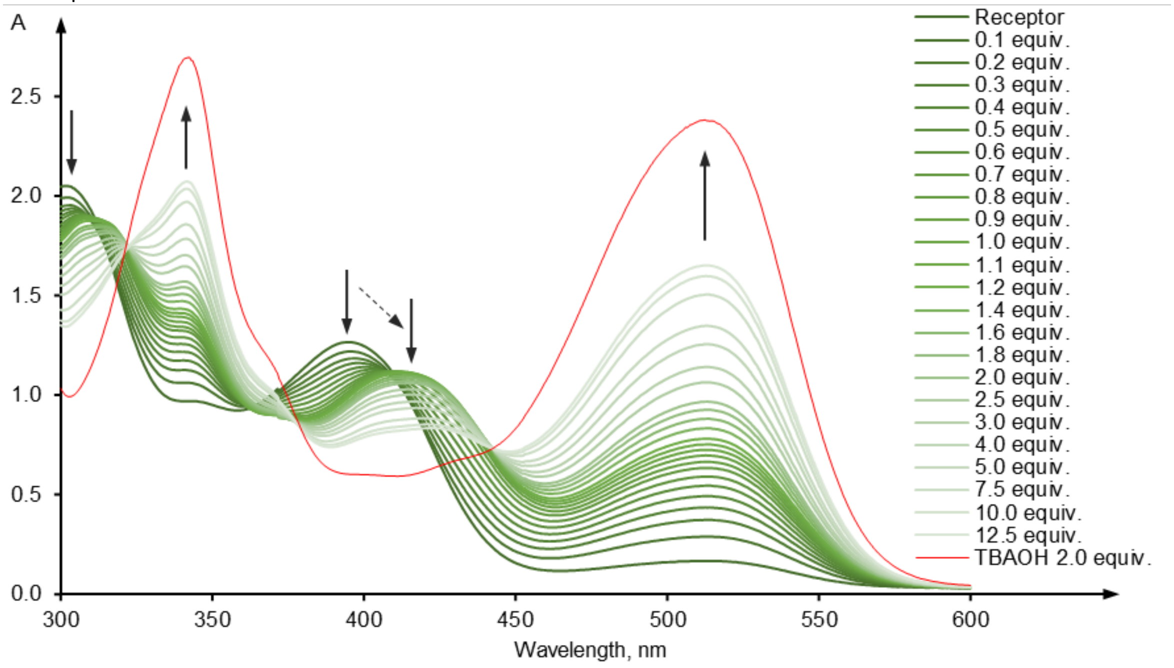

b) Raw data

Equivalents of $TBAH_2PO_4$	343 nm	392 nm	465 nm	513 nm
0.0	0.970	1.261	0.117	0.167
0.1	1.061	1.211	0.183	0.289
0.2	1.123	1.171	0.231	0.373
0.3	1.170	1.140	0.269	0.435
0.4	1.212	1.108	0.305	0.493
0.5	1.249	1.078	0.338	0.545
0.6	1.282	1.050	0.368	0.591
0.7	1.312	1.022	0.397	0.634
0.8	1.331	1.003	0.418	0.665
0.9	1.354	0.986	0.438	0.697
1.0	1.379	0.972	0.455	0.725
1.1	1.398	0.961	0.470	0.752
1.2	1.420	0.952	0.484	0.781
1.4	1.458	0.937	0.509	0.833
1.6	1.495	0.925	0.532	0.881
1.8	1.530	0.914	0.553	0.926
2.0	1.559	0.905	0.572	0.967
2.5	1.635	0.882	0.616	1.064
3.0	1.693	0.863	0.651	1.141
4.0	1.775	0.838	0.702	1.255
5.0	1.848	0.817	0.745	1.348
7.5	1.960	0.781	0.817	1.505
10.0	2.025	0.759	0.861	1.598
12.5	2.064	0.746	0.888	1.652

2.2.3 UV-Vis titration of **4** with $PhCOO^-$ in DMSO/0.5%H_2O

UV-Vis titration of $1{\times}10^{-4}$ M solution of receptor **4** in DMSO/0.5% H_2O with 0.0075 M solution of TBAPhCOO (dissolved in the solution of receptor **4**).

a) UV-Vis spectra

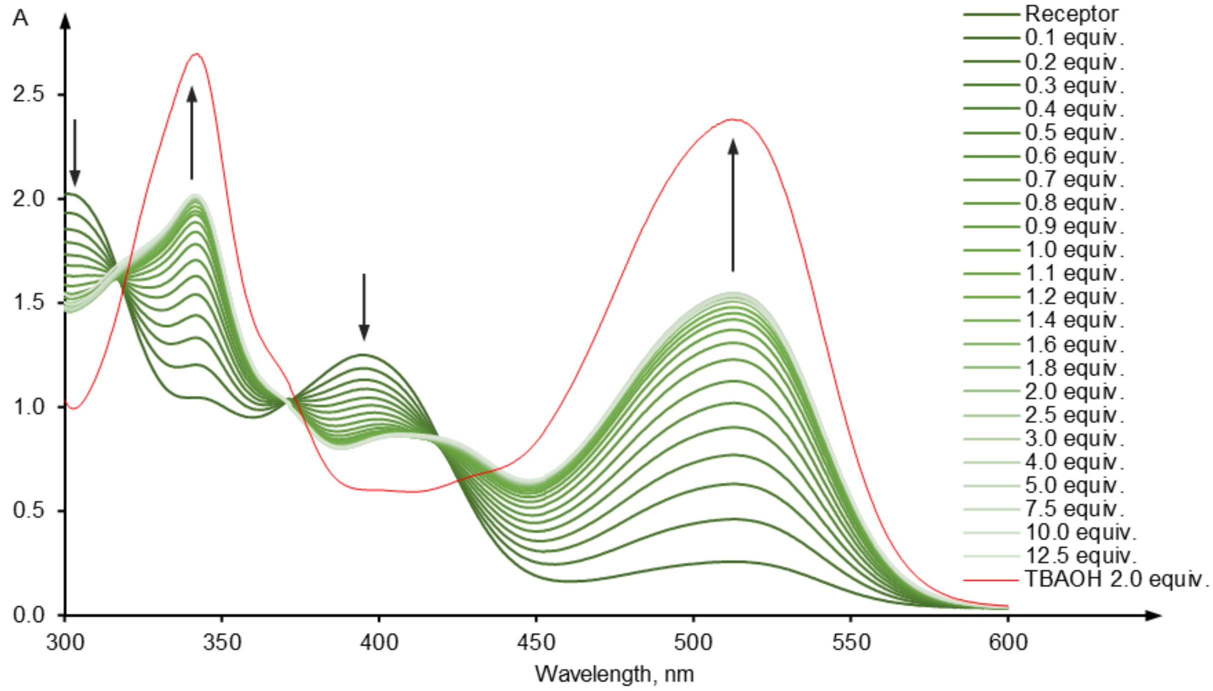

b) Raw data

Equivalents of TBAPhCOO	345 nm	397 nm	450 nm	515 nm
0.0	1.040	1.246	0.189	0.257
0.1	1.189	1.184	0.254	0.460
0.2	1.311	1.130	0.309	0.628
0.3	1.412	1.086	0.356	0.767
0.4	1.509	1.044	0.402	0.900
0.5	1.592	1.008	0.443	1.016
0.6	1.665	0.972	0.480	1.122
0.7	1.737	0.939	0.518	1.225
0.8	1.794	0.912	0.547	1.305
0.9	1.837	0.892	0.571	1.367
1.0	1.871	0.877	0.589	1.416
1.1	1.889	0.868	0.601	1.447
1.2	1.910	0.858	0.613	1.475
1.4	1.930	0.849	0.624	1.504
1.6	1.942	0.844	0.631	1.520
1.8	1.947	0.841	0.635	1.528
2.0	1.947	0.841	0.635	1.528
2.5	1.952	0.842	0.639	1.535
3.0	1.957	0.839	0.642	1.540
4.0	1.960	0.837	0.644	1.543
5.0	1.961	0.837	0.646	1.543
7.5	1.961	0.839	0.648	1.532
10.0	1.961	0.839	0.648	1.532
12.5	1.960	0.846	0.652	1.512

2.2.4 UV-Vis titration of **4** with Cl^- in DMSO/0.5%H_2O

UV-Vis titration of 2×10^{-4} M solution of receptor **4** in DMSO/0.5% H_2O with 0.090 M solution of TBACl (dissolved in the solution of receptor **4**).

a) UV-Vis spectra

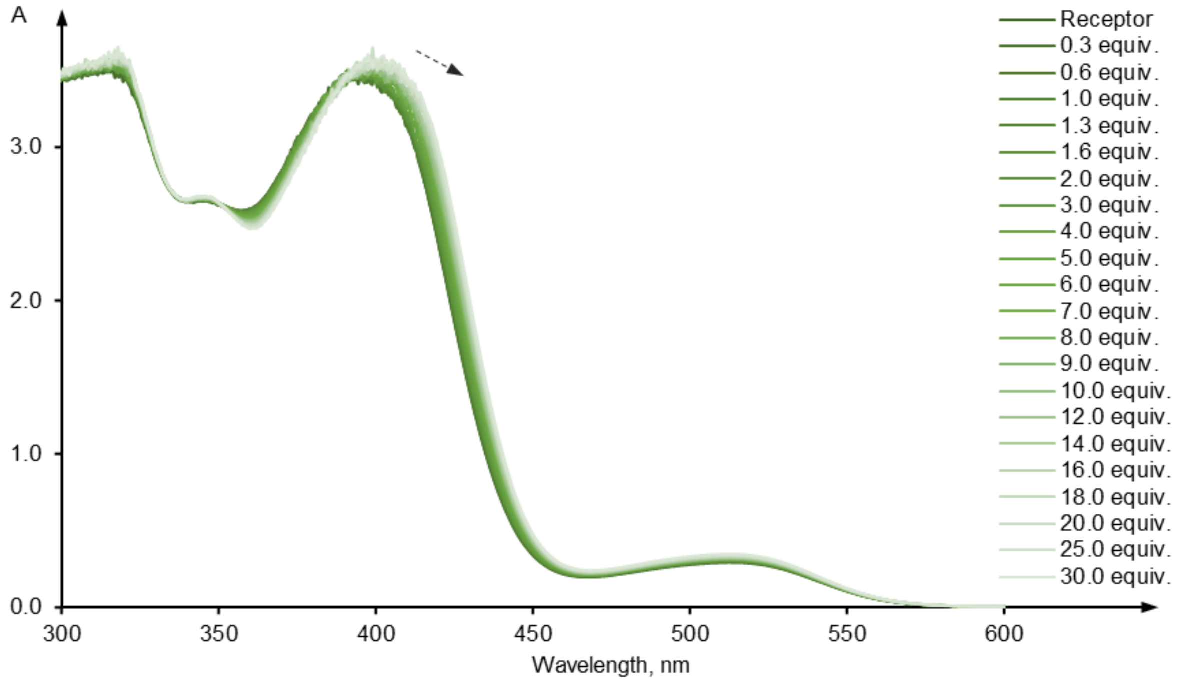

b) Raw data

Equivalents of TBACl	361 nm	430 nm	515 nm
0.0	2.603	1.393	0.291
0.3	2.608	1.403	0.297
0.6	2.606	1.409	0.298
1.0	2.602	1.430	0.300
1.3	2.597	1.455	0.302
1.6	2.597	1.469	0.303
2.0	2.594	1.488	0.305
3.0	2.585	1.516	0.306
4.0	2.572	1.566	0.309
5.0	2.560	1.607	0.310
6.0	2.552	1.647	0.313
7.0	2.543	1.686	0.315
8.0	2.534	1.714	0.315
9.0	2.530	1.742	0.317
10.0	2.519	1.768	0.320
12.0	2.514	1.794	0.322
14.0	2.507	1.835	0.325
16.0	2.499	1.872	0.330
18.0	2.493	1.901	0.333
20.0	2.483	1.927	0.335
25.0	2.480	1.949	0.339
30.0	2.470	1.995	0.346

2.2.5 UV-Vis titration of **4** with OH^{-} in DMSO/0.5%H_2O

UV-Vis titration of 1×10^{-4} M solution of receptor **4** in DMSO/0.5% H_2O with 0.0075 M solution of TBAOH (dissolved in the solution of receptor **4**).

a) UV-Vis spectra

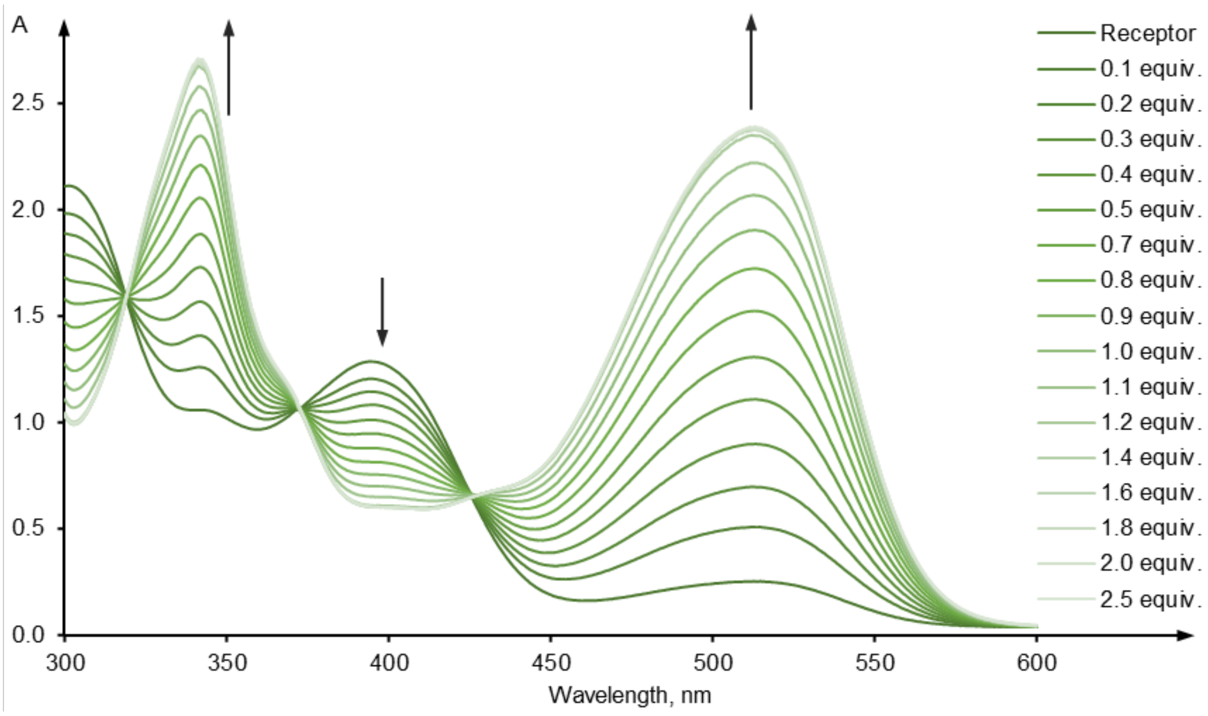

b) Raw data

Equivalents of TBAOH	346 nm	396 nm	450 nm	515 nm
0.0	1.049	1.285	0.190	0.253
0.1	1.231	1.204	0.269	0.508
0.2	1.367	1.144	0.327	0.696
0.3	1.515	1.083	0.388	0.897
0.4	1.662	1.012	0.452	1.107
0.5	1.805	0.948	0.512	1.305
0.6	1.806	0.948	0.512	1.307
0.7	1.961	0.880	0.576	1.522
0.8	2.103	0.814	0.636	1.720
0.9	2.228	0.756	0.690	1.901
1.0	2.340	0.701	0.739	2.064
1.1	2.441	0.650	0.784	2.216
1.2	2.528	0.609	0.823	2.346
1.4	2.550	0.602	0.830	2.372
1.6	2.552	0.602	0.831	2.376
1.8	2.553	0.602	0.831	2.374
2.0	2.554	0.602	0.832	2.377
2.5	2.566	0.604	0.834	2.386

3 Self-dissociation studies

3.1 Self-dissociation studies of receptor **3**

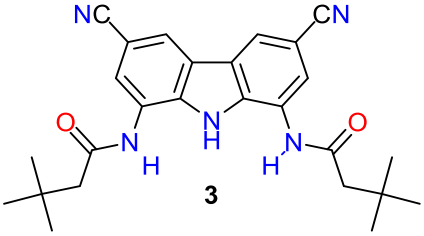

3

UV-Vis titration of 1×10^{-4} M solution of receptor **3** in DMSO/0.5% H_2O with 0.0075 M solution of TfOH (dissolved in the solution of receptor **3**).

a) UV-Vis spectra

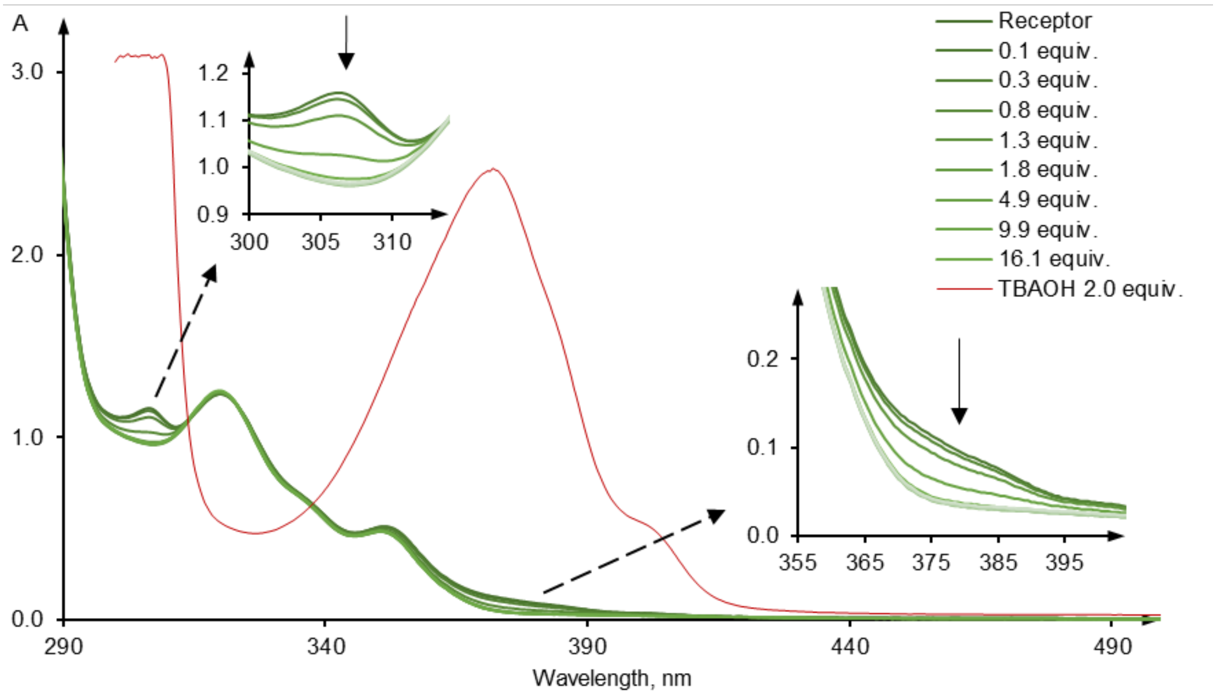

b) Titration curves

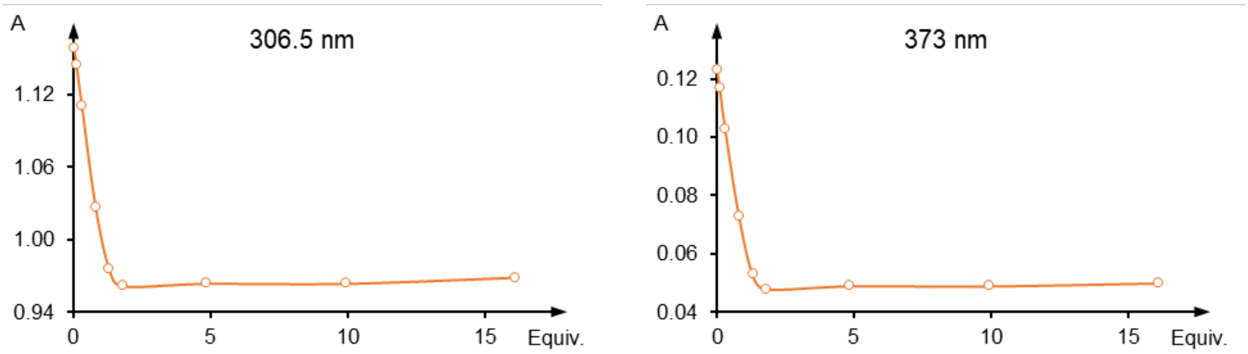

3.2 Self-dissociation studies of receptor **4**

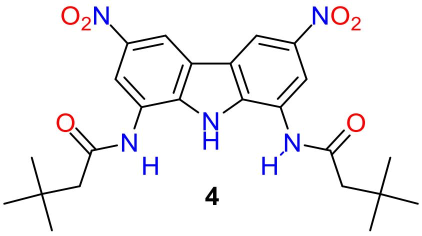

UV-Vis titration of 1×10^{-4} M solution of receptor **4** in DMSO/0.5% H_2O with 0.0075 M solution of TfOH (dissolved in the solution of receptor **4**).

a) UV-Vis spectra

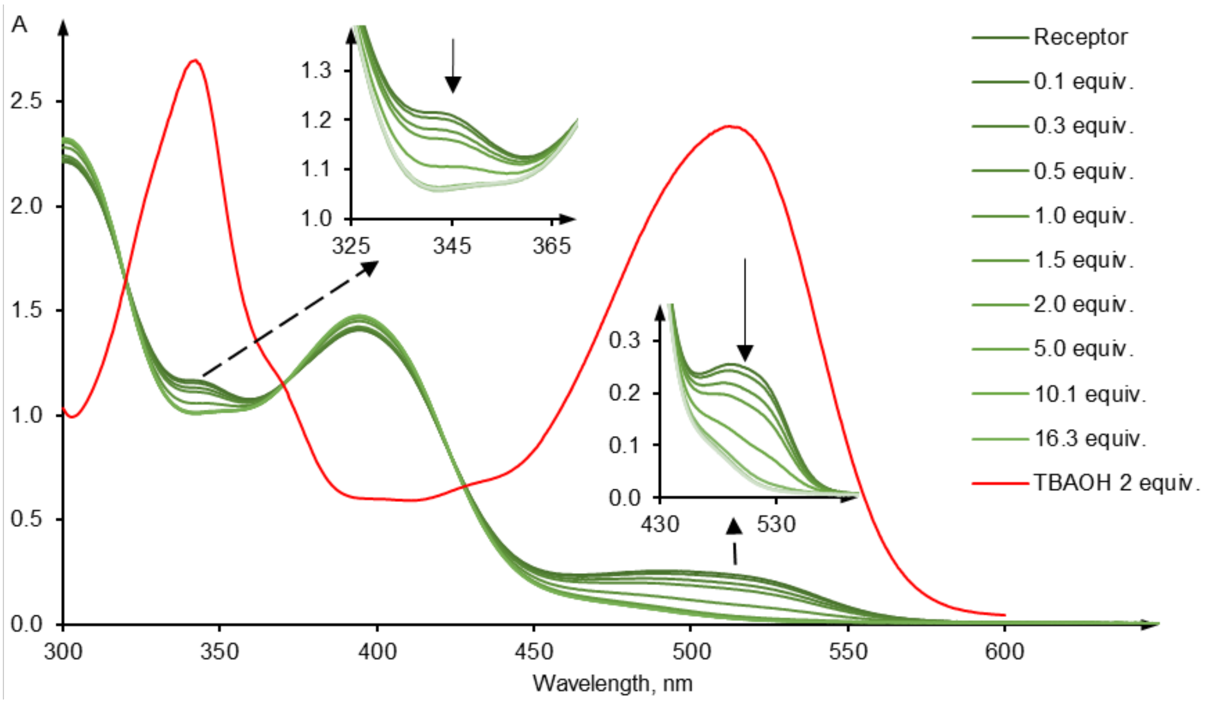

b) Titration curves

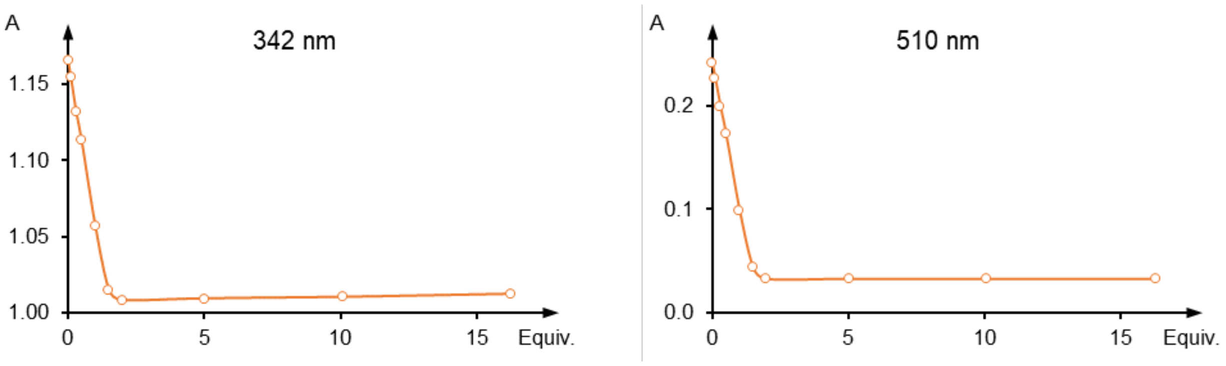

4 Crystallographic data and refinement details

Table S1. Crystal data and structure refinement for investigated compounds.

**Identification code**	**K3 (3)**	**K4 (4)**
CCDC deposition number	2074344	2074345
Empirical formula	$C_{110}H_{122}Cl_2N_{10}O_4P_2$	$C_{53}H_{61}ClN_5O_6P$
Formula weight	1781.01	930.48
Temperature/K	200.00(10)	200(2)
Crystal system	triclinic	triclinic
Space group	P-*1*	P-*1*
a/Å	11.2744(6)	11.1012(3)
b/Å	14.0732(8)	14.3129(5)
c/Å	16.9993(10)	17.1970(5)
α/°	72.779(5)	72.560(3)
β/°	84.390(5)	84.207(2)
γ/°	78.038(5)	76.749(3)
Volume/Å^3	2518.5(3)	2535.90(14)
Z	1	2
ρ_{calc} g/cm^3	1.174	1.219
μ/mm^{-1}	0.153	0.160
F(000)	948.0	988.0
Crystal size/mm^3	0.201×0.104×0.069	0.959×0.216×0.152
Radiation	MoKα (λ = 0.71073)	Mo$K\alpha$ (λ = 0.71073)
2Θ range for data collection/°	4.482 to 52.742	4.326 to 52.744
Index ranges	$-14 \le h \le 14$, $-17 \le k \le 17$, $-21 \le l \le 21$	$-13 \le h \le 13$, $-17 \le k \le 17$, $-21 \le l \le 21$
Reflections collected	37975	42264
Independent reflections	10274 [R_{int} = 0.0651, R_{sigma} = 0.0784]	10365 [R_{int} = 0.0334, R_{sigma} = 0.0309]
Data/restraints/parameters	10274/30/633	10365/1/603
Goodness-of-fit on F^2	1.020	1.067
Final R indexes [I>=2σ (I)]	R_1 = 0.0643, wR_2 = 0.1226	R_1 = 0.0538, wR_2 = 0.1317
Final R indexes [all data]	R_1 = 0.1168, wR_2 = 0.1434	R_1 = 0.0688, wR_2 = 0.1401
Largest diff. peak/hole/e Å^{-3}	0.23/-0.25	0.51/-0.41

Table S2. Bond lengths for complex with **3**.

Atom	Atom	Length/Å	Atom	Atom	Length/Å
C2A	C3A	1.413(4)	C20	C25	1.388(4)
C2A	C7A	1.396(4)	C20	P19	1.794(3)
C2A	N1A	1.373(3)	C21	C22	1.376(4)
C2B	C3B	1.407(4)	C22	C23	1.377(4)
C2B	C7B	1.402(4)	C23	C24	1.380(4)
C2B	N1A	1.369(3)	C24	C25	1.377(4)
C3A	C3B	1.442(4)	C26	C27	1.392(4)
C3A	C4A	1.398(4)	C26	C31	1.390(4)
C3B	C4B	1.392(4)	C26	P19	1.788(3)
C4A	C5A	1.382(4)	C27	C28	1.383(4)
C4B	C5B	1.383(4)	C28	C29	1.374(4)
C5A	C6A	1.406(4)	C29	C30	1.376(4)
C5A	C15A	1.445(4)	C30	C31	1.384(4)
C5B	C6B	1.409(4)	C32	C33	1.383(4)
C5B	C15B	1.443(4)	C32	C37	1.386(4)
C6A	C7A	1.385(4)	C32	P19	1.789(3)
C6B	C7B	1.387(4)	C33	C34	1.382(4)
C7A	N8A	1.403(3)	C34	C35	1.360(5)
C7B	N8B	1.407(3)	C35	C36	1.369(5)
C9A	C10A	1.501(4)	C36	C37	1.382(5)
C9A	N8A	1.369(3)	C38	C39	1.393(4)
C9A	O17A	1.218(3)	C38	C43	1.393(4)
C9B	C10B	1.508(4)	C38	P19	1.789(3)
C9B	N8B	1.366(3)	C39	C40	1.379(4)
C9B	O17B	1.218(3)	C40	C41	1.377(5)
C10A	C11A	1.535(4)	C41	C42	1.371(4)
C10B	C11B	1.544(4)	C42	C43	1.385(4)
C11A	C12A	1.524(5)	C44B	C45B	1.388(10)
C11A	C13A	1.530(4)	C45B	C46B	1.387(11)
C11A	C14A	1.514(5)	C46B	C47B	1.397(11)
C11B	C12B	1.522(4)	C47B	C48B	1.406(11)
C11B	C13B	1.532(4)	C44A	C45A	1.379(13)
C11B	C14B	1.522(4)	C45A	C46A	1.376(14)
C15A	N16A	1.137(4)	C46A	C47A	1.382(14)
C15B	N16B	1.141(3)	C47A	C48A	1.384(13)
C20	C21	1.392(4)			

Table S3. Valence angles for complex with **3**.

Atom	Atom	Atom	Angle/°	Atom	Atom	Atom	Angle/°
C7A	C2A	C3A	123.3(2)	N16B	C15B	C5B	179.7(4)
N1A	C2A	C3A	108.8(2)	C2B	N1A	C2A	109.1(2)
N1A	C2A	C7A	127.9(2)	C9A	N8A	C7A	127.6(2)
C7B	C2B	C3B	122.9(2)	C9B	N8B	C7B	127.9(2)
N1A	C2B	C3B	109.5(2)	C21	C20	P19	120.3(2)
N1A	C2B	C7B	127.6(2)	C25	C20	C21	119.3(3)
C2A	C3A	C3B	106.5(2)	C25	C20	P19	120.3(2)
C4A	C3A	C2A	119.2(3)	C22	C21	C20	120.1(3)
C4A	C3A	C3B	134.3(3)	C21	C22	C23	120.2(3)
C2B	C3B	C3A	106.1(2)	C22	C23	C24	120.1(3)
C4B	C3B	C2B	119.8(3)	C25	C24	C23	120.1(3)
C4B	C3B	C3A	134.1(3)	C24	C25	C20	120.1(3)
C5A	C4A	C3A	117.6(3)	C27	C26	P19	119.6(2)
C5B	C4B	C3B	117.1(3)	C31	C26	C27	119.6(3)
C4A	C5A	C6A	122.7(3)	C31	C26	P19	120.8(2)
C4A	C5A	C15A	120.8(3)	C28	C27	C26	119.5(3)
C6A	C5A	C15A	116.6(3)	C29	C28	C27	120.7(3)
C4B	C5B	C6B	123.3(2)	C28	C29	C30	120.1(3)
C4B	C5B	C15B	119.3(3)	C29	C30	C31	120.1(3)
C6B	C5B	C15B	117.4(3)	C30	C31	C26	120.0(3)
C7A	C6A	C5A	120.8(3)	C33	C32	C37	119.3(3)
C7B	C6B	C5B	120.0(3)	C33	C32	P19	119.5(2)
C2A	C7A	N8A	118.3(2)	C37	C32	P19	120.9(2)
C6A	C7A	C2A	116.5(2)	C34	C33	C32	120.4(3)
C6A	C7A	N8A	125.2(3)	C35	C34	C33	119.7(3)
C2B	C7B	N8B	118.3(2)	C34	C35	C36	120.8(3)
C6B	C7B	C2B	116.8(2)	C35	C36	C37	120.2(3)
C6B	C7B	N8B	124.9(2)	C36	C37	C32	119.6(3)
N8A	C9A	C10A	114.1(2)	C39	C38	P19	119.6(2)
O17A	C9A	C10A	123.1(3)	C43	C38	C39	119.5(3)
O17A	C9A	N8A	122.8(3)	C43	C38	P19	120.8(2)
N8B	C9B	C10B	114.6(2)	C40	C39	C38	120.0(3)
O17B	C9B	C10B	122.9(3)	C41	C40	C39	120.2(3)
O17B	C9B	N8B	122.5(3)	C42	C41	C40	120.3(3)
C9A	C10A	C11A	115.3(2)	C41	C42	C43	120.6(3)
C9B	C10B	C11B	115.6(2)	C42	C43	C38	119.5(3)
C12A	C11A	C10A	110.8(3)	C26	P19	C20	109.55(12)
C12A	C11A	C13A	109.0(3)	C26	P19	C32	109.36(13)
C13A	C11A	C10A	106.6(3)	C26	P19	C38	111.53(13)
C14A	C11A	C10A	110.2(3)	C32	P19	C20	108.10(13)
C14A	C11A	C12A	109.6(3)	C32	P19	C38	109.30(12)
C14A	C11A	C13A	110.6(3)	C38	P19	C20	108.93(13)
C12B	C11B	C10B	111.4(2)	C46B	C45B	C44B	127.2(15)
C12B	C11B	C13B	109.7(3)	C45B	C46B	C47B	133.3(15)
C13B	C11B	C10B	106.7(2)	C46B	C47B	C48B	124.4(13)
C14B	C11B	C10B	110.0(2)	C46A	C45A	C44A	133(2)
C14B	C11B	C12B	109.2(3)	C45A	C46A	C47A	165(3)
C14B	C11B	C13B	109.8(3)	C46A	C47A	C48A	132(3)
N16A	C15A	C5A	177.9(3)				

Table S4. Bond lengths for complex with **4**.

Atom	Atom	Length/Å	Atom	Atom	Length/Å
N1A	C2A	1.372(3)	N15B	O16B	1.218(3)
N1A	C2B	1.374(3)	N15B	O17B	1.223(3)
C2A	C3A	1.408(3)	P19	C20	1.786(2)
C2A	C7A	1.407(3)	P19	C26	1.792(2)
C2B	C3B	1.410(3)	P19	C32	1.796(2)
C2B	C7B	1.406(3)	P19	C38	1.793(2)
C3A	C3B	1.446(3)	C20	C21	1.400(3)
C3A	C4A	1.395(3)	C20	C25	1.389(3)
C3B	C4B	1.393(3)	C21	C22	1.383(4)
C4A	C5A	1.378(3)	C22	C23	1.374(4)
C4B	C5B	1.377(3)	C23	C24	1.381(4)
C5A	C6A	1.399(3)	C24	C25	1.379(3)
C5A	N15A	1.464(3)	C26	C27	1.394(3)
C5B	C6B	1.402(3)	C26	C31	1.390(3)
C5B	N15B	1.464(3)	C27	C28	1.385(4)
C6A	C7A	1.384(3)	C28	C29	1.377(4)
C6B	C7B	1.385(3)	C29	C30	1.375(4)
C7A	N8A	1.405(3)	C30	C31	1.389(4)
C7B	N8B	1.406(3)	C32	C33	1.399(3)
N8A	C9A	1.371(3)	C32	C37	1.395(3)
N8B	C9B	1.368(3)	C33	C34	1.377(3)
C9A	C10A	1.502(3)	C34	C35	1.383(4)
C9A	O18A	1.222(3)	C35	C36	1.386(4)
C9B	C10B	1.510(3)	C36	C37	1.382(3)
C9B	O18B	1.218(3)	C38	C39	1.387(3)
C10A	C11A	1.541(4)	C38	C43	1.391(3)
C10B	C11B	1.542(3)	C39	C40	1.389(4)
C11A	C12A	1.525(4)	C40	C41	1.368(5)
C11A	C13A	1.532(4)	C41	C42	1.379(5)
C11A	C14A	1.521(4)	C42	C43	1.382(4)
C11B	C12B	1.525(3)	C45	C46	1.337(8)
C11B	C13B	1.529(4)	C46	C47	1.414(8)
C11B	C14B	1.522(4)	C47	C48	1.415(9)
N15A	O16A	1.227(3)	C48	C49	1.359(8)
N15A	O17A	1.228(3)			

Table S5. Valence angles for complex with **4**.

Atom	Atom	Atom	Angle/˚	Atom	Atom	Atom	Angle/˚
C2A	N1A	C2B	108.97(18)	C14B	C11B	C12B	109.2(2)
N1A	C2A	C3A	109.40(18)	C14B	C11B	C13B	110.2(3)
N1A	C2A	C7A	127.9(2)	O16A	N15A	C5A	118.23(19)
C7A	C2A	C3A	122.7(2)	O16A	N15A	O17A	122.8(2)
N1A	C2B	C3B	109.09(18)	O17A	N15A	C5A	118.95(19)
N1A	C2B	C7B	127.8(2)	O16B	N15B	C5B	118.6(2)
C7B	C2B	C3B	123.07(19)	O16B	N15B	O17B	122.5(2)
C2A	C3A	C3B	106.18(19)	O17B	N15B	C5B	118.9(2)
C4A	C3A	C2A	120.12(19)	C20	P19	C26	111.18(11)
C4A	C3A	C3B	133.7(2)	C20	P19	C32	109.26(10)
C2B	C3B	C3A	106.36(18)	C20	P19	C38	109.87(11)
C4B	C3B	C2B	119.8(2)	C26	P19	C32	109.38(10)
C4B	C3B	C3A	133.9(2)	C26	P19	C38	109.42(10)
C5A	C4A	C3A	116.1(2)	C38	P19	C32	107.66(11)
C5B	C4B	C3B	116.3(2)	C21	C20	P19	120.01(18)
C4A	C5A	C6A	124.8(2)	C25	C20	P19	120.40(17)
C4A	C5A	N15A	118.3(2)	C25	C20	C21	119.6(2)
C6A	C5A	N15A	116.92(19)	C22	C21	C20	119.5(2)
C4B	C5B	C6B	124.9(2)	C23	C22	C21	120.4(2)
C4B	C5B	N15B	117.9(2)	C22	C23	C24	120.3(2)
C6B	C5B	N15B	117.23(19)	C25	C24	C23	120.2(2)
C7A	C6A	C5A	119.5(2)	C24	C25	C20	120.0(2)
C7B	C6B	C5B	119.3(2)	C27	C26	P19	119.61(18)
C6A	C7A	C2A	116.8(2)	C31	C26	P19	120.50(19)
C6A	C7A	N8A	124.93(19)	C31	C26	C27	119.7(2)
N8A	C7A	C2A	118.29(19)	C28	C27	C26	120.0(3)
C6B	C7B	C2B	116.7(2)	C29	C28	C27	120.0(3)
C6B	C7B	N8B	125.13(19)	C30	C29	C28	120.4(2)
N8B	C7B	C2B	118.21(19)	C29	C30	C31	120.4(3)
C9A	N8A	C7A	127.63(19)	C30	C31	C26	119.5(3)
C9B	N8B	C7B	128.05(19)	C33	C32	P19	119.62(18)
N8A	C9A	C10A	114.26(19)	C37	C32	P19	120.69(17)
O18A	C9A	N8A	122.7(2)	C37	C32	C33	119.6(2)
O18A	C9A	C10A	123.1(2)	C34	C33	C32	119.9(2)
N8B	C9B	C10B	114.81(19)	C33	C34	C35	120.3(2)
O18B	C9B	N8B	122.4(2)	C34	C35	C36	120.2(2)
O18B	C9B	C10B	122.8(2)	C37	C36	C35	120.1(2)
C9A	C10A	C11A	114.8(2)	C36	C37	C32	119.9(2)
C9B	C10B	C11B	115.8(2)	C39	C38	P19	119.31(19)
C12A	C11A	C10A	111.3(2)	C39	C38	C43	120.0(2)
C12A	C11A	C13A	109.1(2)	C43	C38	P19	120.22(19)
C13A	C11A	C10A	106.4(2)	C38	C39	C40	119.9(3)
C14A	C11A	C10A	110.0(2)	C41	C40	C39	119.8(3)
C14A	C11A	C12A	109.5(3)	C40	C41	C42	120.6(3)
C14A	C11A	C13A	110.5(3)	C41	C42	C43	120.4(3)
C12B	C11B	C10B	111.3(2)	C42	C43	C38	119.2(3)
C12B	C11B	C13B	109.5(3)	C45	C46	C47	130.8(8)
C13B	C11B	C10B	106.0(2)	C48	C47	C46	131.5(7)
C14B	C11B	C10B	110.6(2)	C49	C48	C47	130.3(8)

4.1 ^{1}H NMR of crystallised complex [**4** × Ph_4PCl] dissolved in $CDCl_3$

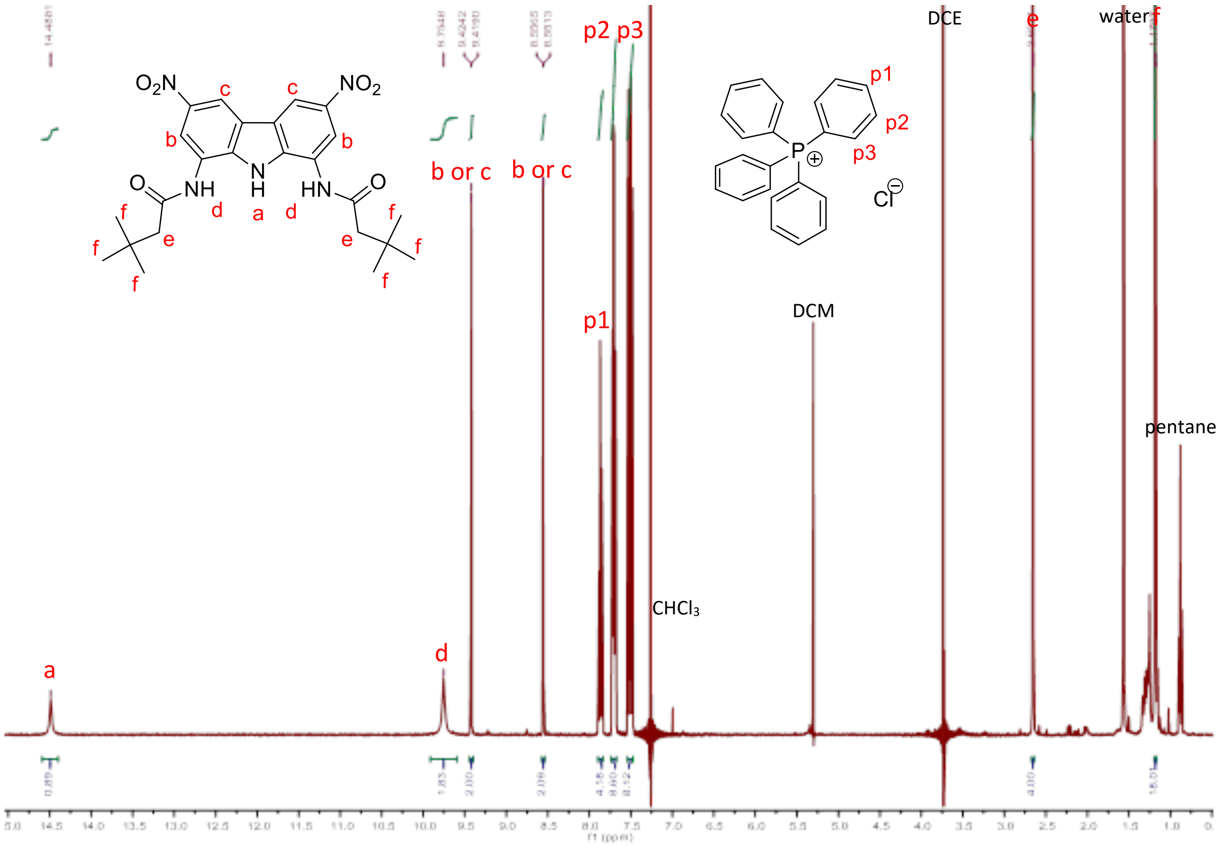

Supplement: Supplementary file 1 [file molecules-26-03205-s001.zip › molecules-1218151-supplementary.pdf]
